# Electrostatic Control of Shape Selection and Nanoscale Structure in Chiral Molecular Assemblies

**DOI:** 10.1021/acscentsci.2c00447

**Published:** 2022-08-02

**Authors:** Joseph M. McCourt, Sumit Kewalramani, Changrui Gao, Eric W. Roth, Steven J. Weigand, Monica Olvera de la Cruz, Michael J. Bedzyk

**Affiliations:** † Department of Physics and Astronomy, Northwestern University, Evanston, Illinois 60208, United States; ‡ Department of Materials Science and Engineering, Northwestern University, Evanston, Illinois 60208, United States; § DuPont-Northwestern-Dow Collaborative Access Team, Northwestern University Synchrotron Research Center, Advanced Photon Source, Argonne, Illinois 60439, United States; ⊥ Department of Chemistry, Northwestern University, Evanston, Illinois 60208, United States

## Abstract

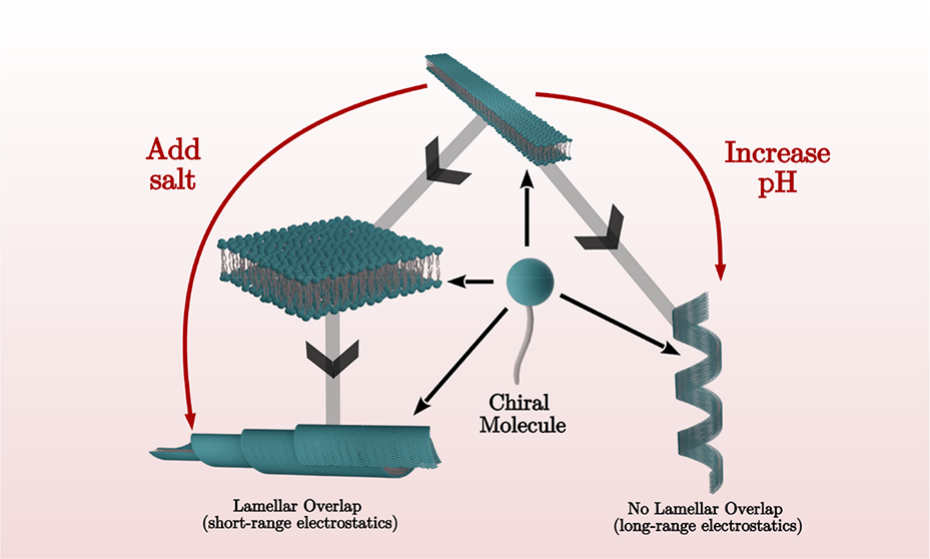

How molecular chirality manifests at the nano- to macroscale has been a scientific puzzle since Louis Pasteur discovered biochirality. Chiral molecules assemble into meso-shapes such as twisted and helical ribbons, helicoidal scrolls (cochleates), or möbius strips (closed twisted ribbons). Here we analyze self-assembly for a series of amphiphiles, C_*n*_-K, consisting of an ionizable amino acid [lysine (K)] coupled to alkyl tails with *n* = 12, 14, or 16 carbons. This simple system allows us to probe the effects of electrostatic and van der Waals interactions in chiral assemblies. Small/wide-angle X-ray scattering (SAXS/WAXS) reveals that at low pH, where the headgroups are ionized (+1), C_16_-K forms high aspect ratio, planar crystalline bilayers. Molecular dynamics (MD) simulations reveal that tilted tails of the bilayer leaflets are interdigitated. SAXS shows that, with increasing salt concentration, C_16_-K molecules assemble into cochleates, whereas at elevated pH (reduced degree of ionization), helices are observed for all C_*n*_-K assemblies. The shape selection between helices and scrolls is explained by a membrane energetics model. The nano- to meso-scale structure of the chiral assemblies can be continuously controlled by solution ionic conditions. Overall, our study represents a step toward an electrostatics-based approach for shape selection and nanoscale structure control in chiral assemblies.

## Abstract 2

Electrostatic interactions dictate chiral shape selection between scroll-like cochleates and helical ribbons in charged molecular assemblies.

## Introduction

**Figure 1:**
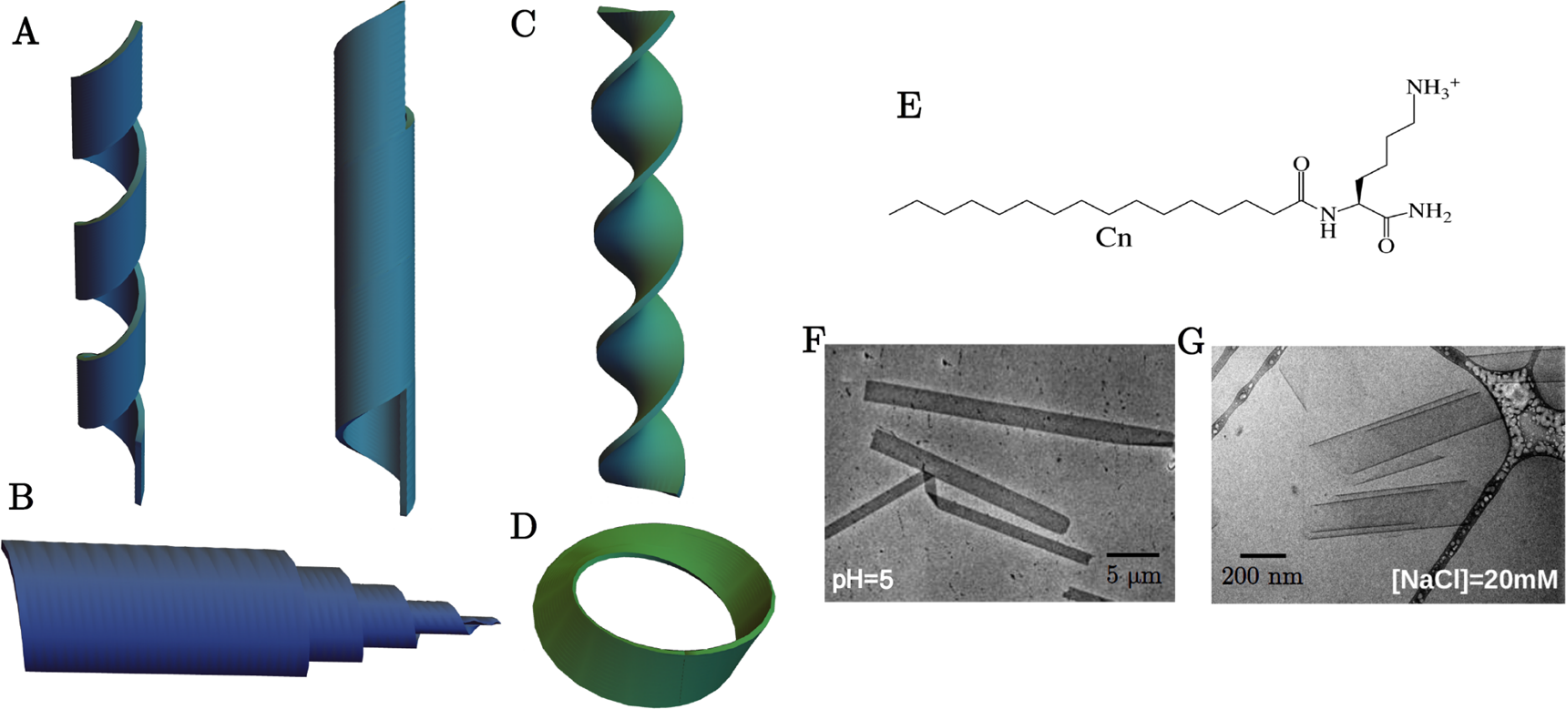
Schematics of chiral shapes: (A) helical ribbon and closed helical tubule, (B) cochleate (scroll-like), (C) twisted ribbon, and (D) möbius strip. (E) Schematic for the molecular design for the homologous series of amphiphiles C_*n*_-K. (F) TEM image of L-C_16_-K flat ribbons and (G) cryo-TEM cochleate structure for L-C_16_-K in [NaCl] = 20 mM.

Chiral molecules are ubiquitous in biology and synthetic chemistry. Examples include amino acids that constitute the proteins, lipids that constitute the cell membranes, and synthetic peptide amphiphiles. Molecular chirality is often manifested in fascinating mesoscopic chiral shapes (Figure 1A–D) such as helical ribbons and nanotubes with barber-pole-like markings,^1−8^ twisted ribbons,^7−11^ helicoidal scrolls (cochleates),^12−15^ and möbius strips.^16^ Self-assembly in simple synthetic chiral molecular systems can provide insights into important biophysical processes. For example, helical ribbons and tubules observed in synthetic bile salt are analogous to chiral shapes observed in gallstone formation.^17^ Furthermore, these soft chiral assemblies have potential nanotechnological applications that depend sensitively on the overall shape and nm-scale structural details of these assemblies. For example, helicoidal scrolls are being explored as drug/macromolecular delivery platforms due to their ability to encapsulate nanoscale objects within the bilayers (hydrophobic molecules) and in the aqueous phase between adjacent bilayers (hydrophilic molecules).^13^ Here, the bilayer thickness and the interbilayer separation should determine the size of the objects that such cochleates can trap and release. Helical ribbons and nanotubes are recognized as possible templates for nano- and meso-electronic components such as nanowires and solenoids.^18,19^ Clearly, the diameter and the helical pitch of these assemblies determine the nanowire properties and solenoid turn densities. For applications of helices and nanotubes, see excellent reviews, refs (4, 5, and 20), and references therein. These examples illustrate the need for developing control over shape selection, internal architecture of chiral assemblies, and interconversion mechanisms.

For membranes the origin of chiral shapes lies in the out of plane bending force that arises because a close packing of chiral molecules necessitates a relative twist between the neighboring molecules. This is qualitatively analogous to the case of packing of hard screws of a given handedness.^21^ Theoretical studies based on continuum elasticity models show that simultaneous constraints of a preferred molecular tilt with respect to the membrane surface and the chirality-induced twist stabilize curved or bent membrane shapes such as open or closed helices,^22−24^ twisted ribbons^9^ and scrolls.^15^ While theoretical models include the tilt ordering, they exclude positional correlations, including crystallinity in molecular packing. This is despite experimental hints of an intricate coupling between molecular packing and chiral assembly. For example, a spherical vesicle is the equilibrium morphology for diacetylenic phospholipid membranes in the high temperature fluid phase (molten lipid tails, L_α_ phase). By contrast, these same membranes in the low temperature condensed phase (tightly packed tilted lipid tails, L_β′_ phase) bend into chiral tubules.^25^ Similarly, the assembly of a peptide amphiphile (*N*-α-lauryl-lysyl-aminolauryl-lysyl-amide) exhibits transformation from helical membranes to achiral spherical micelles when the temperature is raised above the chain melting transition temperature.^26^ However, the effect of membrane fluidity or crystallinity on chiral assemblies is an unresolved issue.^24^ In this context, the current study deals with chiral assemblies of crystalline membranes.

Attractive and repulsive interactions determine the details of molecular packing, and thereby indirectly modulate chiral assemblies. To illustrate, recent experimental studies have shown that electrostatic interactions can have profound effects on the chiral shape selection and structures. For example, zwitterionic phospholipids assemble into helices and nanotubes.^2,3,25^ By contrast, cochleates have been observed for phospholipids that are negatively charged.^12−14^ Similar is the case for peptide amphiphiles with ionizable amino acids:^27^ tuning the molecular charge can induce a transformation between helical and twisted ribbons.^28^ Furthermore, the range of electrostatic interactions controls the twist-pitch in amyloid peptide fibrils.^29^

Despite the above-described progress, a clear understanding of the interconversion mechanisms between different chiral shapes and controls for nanoscale structures of chiral morphologies are lacking. This knowledge gap is likely due to a dearth of suitable molecular systems and theoretical models that enable exploration of the phase space of chiral shapes by systematically tuning the important intermolecular interactions. To address this, we designed a homologous series of amphiphiles C_*n*_-K (Figure 1E) consisting of a single ionizable, chiral amino acid headgroup (lysine, K) that is covalently coupled to alkyl tails of varying lengths (*n*). We note here that lysine and polylysine amphiphiles, in particular, C_16_-K_*n*_ (*n* = 1–3), have been explored in the context of antimicrobial properties.^30^ However, in that study,^30^ no structural analysis of C_16_-K assemblies was performed. The simple molecular design of C_*n*_-K in the present work allows control over intermolecular electrostatic, van der Waals, and chiral interactions. For electrostatic interactions, the molecular charge can be tuned via pH. For very dilute solutions, the fraction of headgroups that are ionized (degree of ionization, ) is expected to decrease with increasing pH according to , where *K*_a_ is the reaction coefficient for the deprotonation of the lysine headgroup. The range of electrostatic interactions (screening length λ_*D*_) can be controlled by salt concentration *c* via the electrostatic potential, which is of the form ; . We note that, in the very low salt concentration regime, the interaction is dominated by the long-range Coulomb potential *V*(*r*) ∝ 1/*r*, and there is no theoretical work on charged chiral morphologies in this regime. The strength of the attractive van der Waals interactions can be tuned by the alkyl tail length, and in principle, the strength of the chiral interactions can be altered by producing binary mixtures with varied ratios of molecules with right- (*D*) and left-handed (*L*) lysines.

As a part of analyzing chiral assemblies by systematically varying the intermolecular interactions in the C_*n*_-K molecular series, we recently reported on the C_16_-K assembly behavior as the range of intermolecular electrostatic interactions λ_*D*_ was tuned from ∼10 to 1 nm by addition of salt ([NaCl] = 0.001–0.1 M).^15^ This study showed that under conditions where nearly all the lysines were expected to be ionized (+1, pH ≪ p*K*_a_), C_16_-K molecules formed high aspect ratio (*L*/*W* > 10), flat, crystalline bilayers. These bilayer ribbons transformed to sheets (*L*/*W* ∼ 1), which rolled up into helicoidal scrolls as the NaCl concentration was increased. Furthermore, the interbilayer spacing in the scrolls varied linearly with λ_*D*_.^15^ These results are reproducible, as demonstrated by transmission electron microscopy (TEM) images of the high aspect nanoribbons and cochleates observed in the newly synthesized batch of C_16_-K that is used in the present work (Figure 1F,G). Here, we extend this work to analyze how the coupling between electrostatic and van der Waals interactions controls the chiral shape selection and internal structure. Specifically, we analyze assemblies for *n* = 12, 14, and 16 molecules as a function of the solution pH, that is, the average molecular charge. To be explicit, our previous report focused on enhancing the effects of chiral interactions by screening the intermolecular electrostatic interactions. In this work, the strength of electrostatic interaction is reduced by lowering the degree of ionization of the molecular headgroups, but the electrostatic interactions remain long-ranged.

## Results and Discussion

We first describe and discuss the assembly behavior of C_16_-K as a function of solution pH. Thereafter, the generality of these findings is tested through the characterization of C_12_-K and C_14_-K assemblies.

### Relationship between pH and Degree of Ionization for C_16_-K

**Figure 2:**
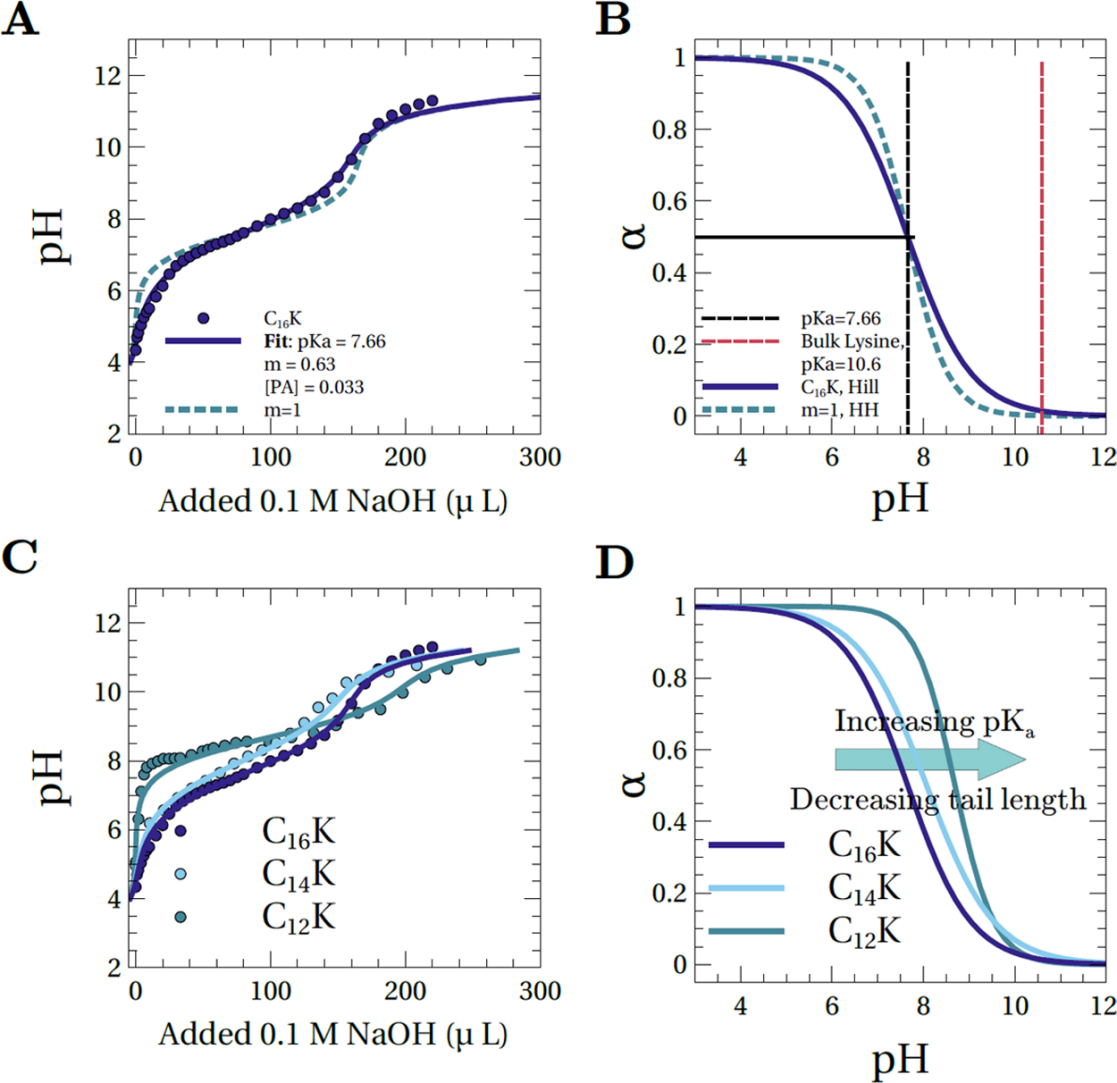
(A) pH titration curve for a 4 mM L-C_16_-K solution in 5 mL of pure water with a 0.1 M NaOH solution (circles) along with a fit based on the empirical Hill equation (solid blue curve). For comparison, also shown is a simulation with the Henderson–Hasselbalch (HH) equation (dashed light blue curve), with a p*K*_a_ that is identical to that derived from the Hill equation fit. (B) Degree of ionization (α) vs pH, as derived from the fits based on the Hill equation (solid blue) and the HH equation (dashed light blue). As a reference, the p*K*_a_ values for L-C_16_-K and free lysine are shown (dashed black and red lines, respectively). (C) Measured titration curves for 4 mM C_*n*_-K in 5 mL of pure water with 0.1 M NaOH. The data points are shown in circles and the Hill equation fits in solid lines. (D) The degree of ionization vs pH for C_*n*_-K that are deduced from the Hill model fits to the pH titration curves in (C).

To quantitatively relate pH and the degree of ionization (α), we titrated 5 mL of a 4 mM L-C_16_-K solution in pure water with a 0.1 M NaOH solution (Figure 2A). This titration curve could be modeled with the empirical Hill equation (eq 1, see also SI, section 2),^31^ which is a modified form of the Henderson–Hasselbalch (HH) equation (eq 1, *m* = 1 case) that works well for dilute solutions.
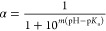
1

In eq 1, p*K*_a_ represents the center of the narrow pH window for the deionization of the molecular headgroups [α(pH = p*K*_a_) = 0.5]. The parameter, *m*, determines the rate of change of the degree of ionization with pH. Therefore, *m* also determines the width of the aforementioned pH window. The best-fit values, p*K*_a_ = 7.66 and *m* = 0.63 (Figure 2A), indicate strong deviations from the dilute solution behavior and suggest that C_16_-K molecules form tightly packed assemblies. To be explicit, (1) the fit value of *m* = 0.63 differs significantly from *m* = 1 (HH). This implies that the ionization and deionization of distinct molecular headgroups are not independent events. In particular, *m* < 1 reflects anticooperativity between molecules with regard to existing in identical ionization states.^31^ (2) The p*K*_a_ = 7.66 is significantly different from the p*K*_a_ = 10.54 for free lysines.^32^ This nearly 3 orders of magnitude shift in the acid–base equilibrium constant, which is qualitatively consistent with observations on assemblies of other charged amphiphile molecules,^33,34^ implies a strong reduction in the tendency of the lysines in C_16_-K to be ionized. Such charge regulation is expected because any arrangement of like-charged molecules in proximity increases the electrostatic potential energy of assemblies. The sought-after degree of ionization versus pH curve, which is derived from the best-fit parameters for the Hill equation, is shown in Figure 2B

### Chiral Assembly for C_16_-K at Elevated pH

**Figure 3:**
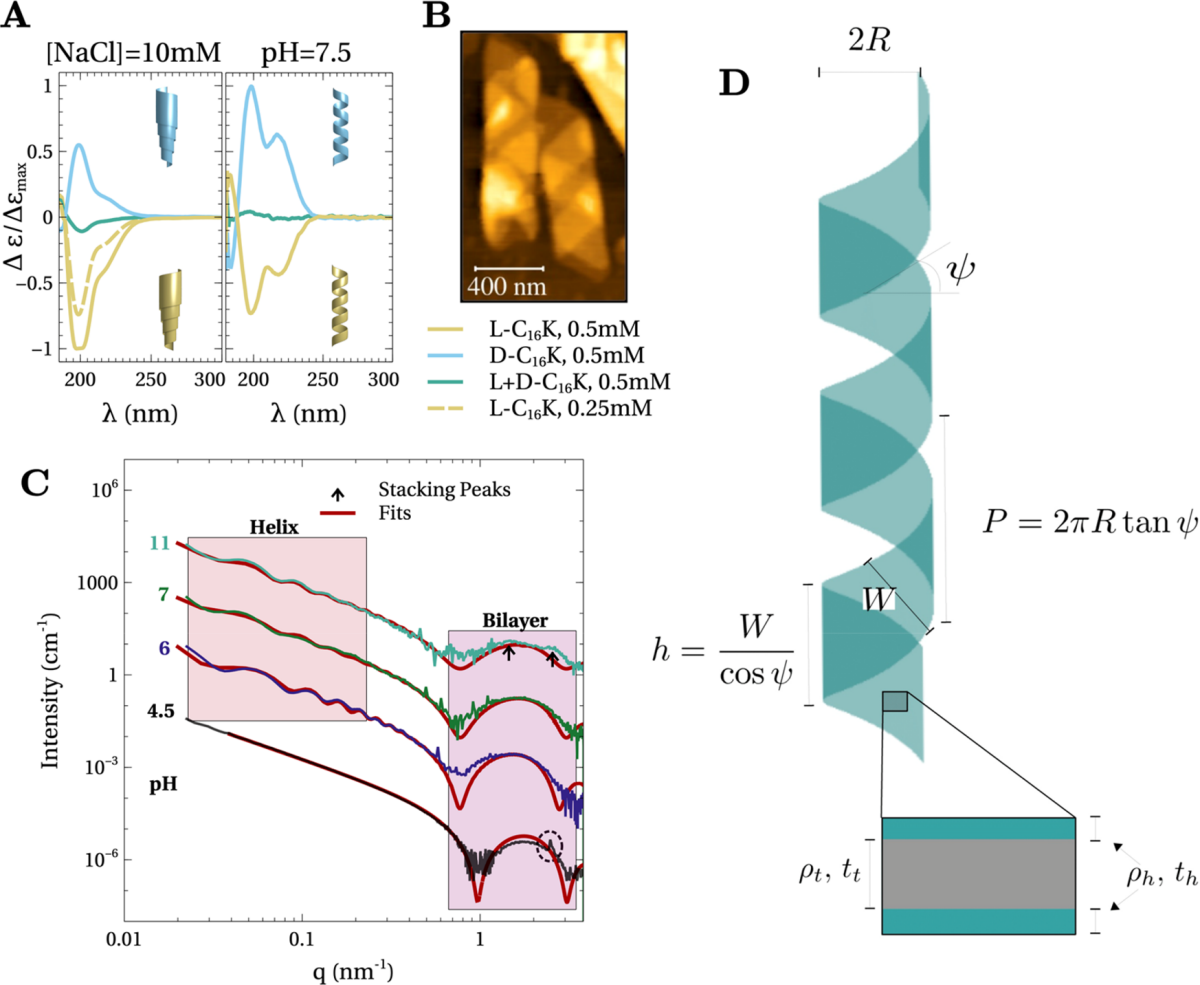
(A) Circular dichroism spectra for *C*_16_-K molecular assemblies in solutions with (1) pH ∼ p*K*_a_ (right) and (2) 10 mM NaCl and pH ≪ p*K*_a_ (left). Insets show schematics of the chiral shapes derived from Cryo-TEM or AFM measurements. (B) Ex situ AFM image of C_16_-K assemblies at pH ∼ 8.5 that were drop-cast and dried on a Si (1 0 0) substrate. (C) In situ SAXS intensity profiles for 4 mM *L*-C_16_-K assemblies in solutions at 4 different pH. The data for pH 6, 7, and 11 are scaled for clarity. Also shown are fits (red line) based on a planar bilayer (pH = 4.5) or helix (pH= 6, 7, 11) models. (D) Schematics of the helix model used to fit data. Left side shows a bilayer membrane that is twisted into a helix parametrized by three independent parameters. For example, the radius (*R*), the helix angle (ψ), and the width (*W*). Right side shows a cross-section of the bilayer membrane.

To test that the strategy of reducing the degree of ionization of the molecular headgroups leads to chiral assemblies, we performed circular dichroism (CD) spectroscopy on 0.5 mM solutions of right- (*D*) or left-handed (*L*) enantiomers or a racemic mixture of C_16_-K (Figure 3A, right). For these measurements, pH ∼ p*K*_a_ (α ∼ 0.5, Figure 2B). In Figure 3A, Δε = ε_L_ – ε_R_, where ε_L_ (ε_R_) is the molar absorption coefficient for the left (right) circularly polarized light, and λ is the wavelength of light. The clearly observable CD signals for both *L*- and *D*-C_16_-K in Figure 3A, right, show that the assemblies at pH ∼ p*K*_a_ are chiral. This contrasts with the flat crystalline bilayers observed at low pH ≪ p*K*_a_ (Figure 1F). Furthermore, the handedness of these chiral assemblies is determined by the enantiomeric form of the molecules because the CD signals have opposite signs for *L*- and *D*-C_16_-K. The CD signal is nearly zero at all wavelengths for the racemic mixture (Figure 3A, right). The zero CD signal for the racemic mixture implies that either (1) the molecules phase-segregated such that each assembly consisted of molecules of only a specific handedness, that is, the solution consisted of an equal number of right- and left-handed assemblies, or (2) the right- and left-handed molecules coassembled to form achiral assemblies. SAXS measurements (SI, section 3) revealed that the latter case holds true in the present study. In particular, the molecules assemble into stacks of flat bilayers for the racemic mixture. Finally, the CD spectra for *L*- and *D*-C_16_-K is characterized by an absorption doublet at λ ∼ 200 and 220 nm (Figure 3A, right).

Atomic force microscopy (AFM, Figure 3B) of a drop-cast 4 mM *L*-C_16_-K, pH = 8.5 (>p*K*_a_) solution onto a Si (0 0 1) substrate showed right-handed helices of diameter ∼250–300 nm (e.g., Figure 3B). Taken together, CD spectroscopy and AFM observations imply that the *L*-C_16_-K (*D*-C_16_-K) flat bilayer ribbons twist into right- (left-) handed helices when the molecular degree of ionization is reduced by increasing the solution pH.

We note that the same correlation between the molecular enantiomeric form and the handedness of the assemblies was deduced for helicoidal scrolls formed in saline solutions by combining CD spectroscopy (Figure 3A, left) and AFM^15^ and cryo-TEM (Figure 1G). Previous theoretical^35^ and experimental^36^ studies have shown that molecules of a given enantiomeric form can assemble into either right- or left-handed meso-shapes. This selection is determined by the coupling between the chiral interactions and the direction of the molecular tilt.^35^ Cochleates (Figure 1G) and helices (Figure 3B) originate from the same C_16_-K bilayer membranes (Figure 1F) in distinct ionic conditions. Therefore, the observation of the same handedness of the meso-shapes for a given molecular enantiomeric form is expected. The key result here is that achiral electrostatic interactions strongly affect chiral shape selection. Reducing the screening length by adding salt leads to cochleates. By contrast, reduction in the strength of electrostatic interactions through controlling the degree of ionization produces helices.

Finally, we note that temperature-dependent CD spectroscopy (SI, section 4) suggests that ordering/crystallinity in molecular packing is essential for the formation of the observed mesoscopic chiral shapes. In particular, the CD signal for helicoidal scrolls (Figure 3A, left) vanishes above *T* ∼ 60 °C (SI, section 4), which is within the observed range of chain melting transition temperatures for C_16_ tails that are coupled to charged headgroups.^37^ This requirement of crystalline packing of alkyl tails is consistent with the examples of diacetylenic phospholipid^25^ and peptide amphiphile (*N*-α-lauryl-lysyl-aminolauryl-lysyl-amide)^26^ membranes discussed above. The details of the molecular packing for C_*n*_-K membranes are deduced via X-ray scattering and MD simulations (discussed later).

### Evolution of C_16_-K Assembly Structure with pH

In situ small-angle X-ray scattering (SAXS) was utilized to analyze the structural evolution of the C_16_-K assemblies as a function of pH. Figure 3C shows the background-subtracted SAXS intensity profiles as a function of the scattering vector magnitude  for 4 mM *L*-C_16_-K at pH = 4.5, 6, 7, and 11, which correspond to α = 0.99, 0.92, 0.72, and 0.008, respectively. Here, λ is the X-ray wavelength and Θ is one-half of the angle between the incident and the scattered X-rays.

**Figure 4:**
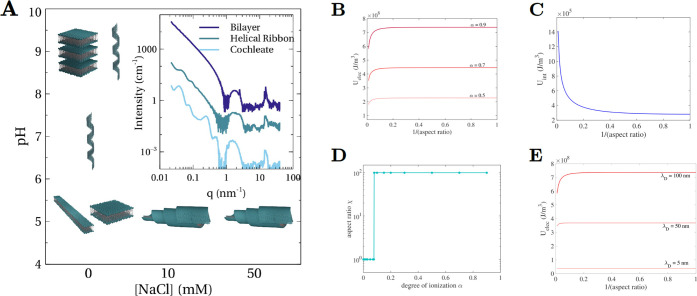
(A) Structural phase diagram for C_16_-K assemblies as a function of salt concentration and pH. The inset shows the characteristic SAXS/WAXS intensity profiles for planar bilayers, helices, and cochleates. Note that the SAXS patterns from the cochleates show diffraction peaks in the SAXS regime from membrane stacking (*q* ∼ 0.25 and ∼0.5 nm^–1^ in the above example). (B) The variation of the rectangular membrane electrostatic energy density as a function of aspect ratio χ for different degrees of ionization α at a fixed λ_*D*_ = 100 nm. (C) Interfacial energy density as a function of χ. (D) Aspect ratio corresponding to the minimum total membrane energy as a function of α. (E) Membrane electrostatic energy density as a function of χ for three different screening lengths (λ_*D*_), which correspond to the cases of NaCl concentrations of ∼10 μM, 40 μM, and 5 mM (top to bottom). For these calculations, α = 1. The numerical calculations (B–E) were carried out for aspect ratio range: 100 ≥ χ ≥ 1, which encompasses the experimentally observed range (χ ∼ 10–30) for bilayer ribbons (example, Figure 1E). For degree of ionization >∼0.07, the membrane energy was found to be minimized for the highest χ (=100) used in the calculations (D).

For pH = 4.5 (Figure 3C, bottom), the intensity profile is consistent with planar, interdigitated bilayers with crystalline ordering in the packing of the molecular headgroups and tails. This is because (1) for low *q* (<0.4 nm^–1^), the scattered intensity drops off monotonically as *I*(*q*) ∝ *q*^–2^. The Porod exponent of −2 is consistent with planar objects (Figure 1F) with both the lateral dimensions larger than .^38^ Here, *q*_min_ is the smallest accessible *q* in our measurements. (2) Fitting the intensity profile with a bilayer model^39^ reveals that the broad intensity modulation in the 0.8 nm^–1^ < *q* < 3 nm^–1^ is due to a 3.82 nm thick bilayer. The thicknesses of the hydrophobic tail and the hydrophilic headgroup regions were determined to be *t*_t_ = 2.30 nm and *t*_h_ = 0.76 nm. (3) The intensity profile shows sharp diffraction peaks in the SAXS (at *q* ∼ 2.5 nm^–1^, Figure 3C, dashed black circle) and the wide-angle X-ray scattering [(WAXS), *q* > 10 nm^–1^] regimes (Figure 4A, inset). These diffraction peaks originate from crystalline ordering in the packing of the headgroups and molecular tails. We note that the expected length for a C_16_ alkyl tail in stretched *trans*-configuration is (16 – 1) × 0.127 nm ∼ 1.9 nm.^40^ Therefore, *t*_t_ = 2.3 nm is substantially lower than the expected length of 1.9 × 2 nm for two C_16_ tails. This is due to the interdigitation of the tails from the two bilayer leaflets, as demonstrated by our molecular dynamics (MD) simulations (discussed later).

**Figure 5:**
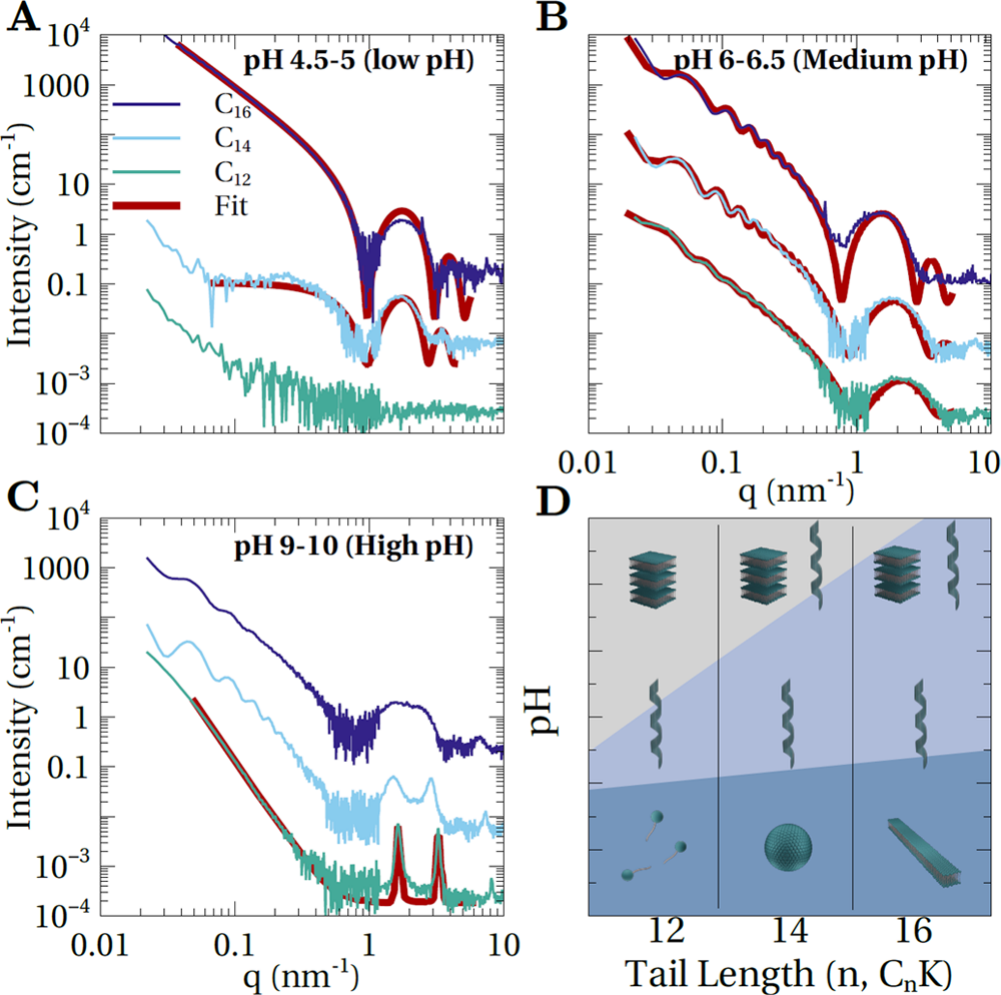
SAXS intensity profiles for 4 mM C_*n*_-K solutions at low (A), medium (B), and high (C) pH. The data is shown along with fits (red solid lines) for all the cases where the solution consisted of the assemblies of a single type. Fit parameters for helical ribbons are listed in Table 3. Based on multibilayer model,^3,9^ the fitting of high pH SAXS data for C_12_-K (C) yielded *N* = 140 for number of lamella. (D) SAXS-derived structural phase diagram for C_*n*_-K assemblies as a function of molecular tail length (*n* = 12, 14, 16) and pH.

In contrast to the planar bilayer case (pH = 4.5), the intensity profiles at elevated pH show multiple modulations in the low *q* (<0.8 nm^–1^) region (Figure 3C). For helical bilayer ribbons, the period of these modulations is primarily determined by the helix radius *R*. Furthermore, for a given *R*, the absolute positions and the amplitude of these modulations depend sensitively on the pitch angle ψ and the ribbon width *W* (SI, section 5). Additionally, for the pH = 11 case, where most of the molecules are expected to be in the deionized state (α = 0.008), the intensity modulation due to bilayer thickness (0.8 nm^–1^ < *q* < 3 nm^–1^) exhibits a nearly flat top with two shallow maxima (weak diffraction peaks, Figure 3C, black arrows). These diffraction peaks arise due to membrane stacking. Therefore, we interpret that the C_16_-K assembly at pH = 11 comprises of a mixture of multiple stacks of bilayers and helical ribbons. This reorganization of membranes into multilamella will become apparent when discussing later the assembly in C_12_-K and C_14_-K as for those cases, the stacking peaks are pronounced in the scattering profiles (Figure 5C). These observations imply that C_16_-K assemblies transform from isolated high aspect ratio bilayers to helices to stacked membranes as the degree of ionization is reduced via pH.

We first ignore the stacking aspect and describe the fitting of the SAXS intensity profiles at pH = 6, 7, and 11 based on a helical bilayer membrane model depicted in Figure 3D. The scattered intensity from helices is distributed as cylindrical Bessel functions on reciprocal space planes defined by .^41^ Here, *q*_∥,*n*_ is the scattering vector component parallel to the helix axis and *P* is the helix pitch. We have analyzed the measured scattered intensity from helical ribbons using the multilayer helical membrane form factor,^42^ which is an extension of the Pringle and Schmidt model.^41,43^ Furthermore, we have taken into account polydispersity in helix size (eqs 2 and 3).
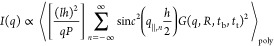
2Here,
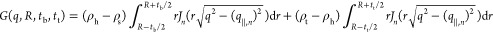
3In eq 2, *l* is the number of turns in the helical bilayer membrane, *h* is the membrane width along the helix axis, *t*_b_ is the bilayer thickness and *t*_t_ is the thickness of the hydrophobic tail region (Figure 3D). In eq 3, ρ_h_ and ρ_t_ are the electron densities for the headgroup and hydrophobic tail regions of the amphiphilic bilayer, ρ_s_ is the solvent electron density, *R* is the mean radius, *J*_*n*_ are the *n*^th^ order Bessel functions of the first kind and  is the scattering vector component normal to the helix axis. Size dispersity (eq 2) is taken into account by averaging over bilayer helical ribbons of 10 different equally spaced radii in the range *R* ± (% polydispersity)/100)*R*. Finally, the summation in eq 2 was found to converge through inclusion of terms within *n* = ±3. For other fitting procedure details, see section 6, SI.

Figure 3C shows the measured intensity profiles along with fits based on the helical bilayer membrane model (eqs 2 and 3). The best fit parameters are listed in Table 1. This analysis shows that the helix radius increases monotonically as the degree of ionization is reduced by increasing the pH. Note that the SAXS-extracted helix radii (Table 1) are smaller than the AFM-derived radii (Figure 3B) by a factor of 1.5–2. This is likely because the dried-out helices in the ex situ AFM measurements were in a collapsed state. This collapse effect has been observed also for phospholipid helices and nanotubes.^2^ If the helices completely flatten on drying, then the apparent radius in AFM is expected to be larger than the real radius by a multiplicative factor of π/2.^2^ This explains the discrepancies between our SAXS and AFM measurements.

**Table 1:** SAXS-Derived *L*-C_16_-K Helix Parameters^a^

pH	*R* (nm)	ψ (deg)	*W* (nm)	*h*/*P*	% polydispersity
6	62.6	29.3	115.6	0.59	5.5
7	69.6	31.9	128.1	0.56	8.7
11	78.2	39.5	189.3	0.61	10.7

a For parameter definitions, see Figure 3D. The case *h*/*P* = 1 corresponds to nanotubes with helical markings, and *h*/*P* < 1 corresponds to open helices. Bilayer parameters were fixed as *t*_h_ = 0.75 nm, *t*_t_ = 2.3 nm, ρ_h_ = 430 e/nm^3^, and ρ_t_ = 307 e/nm^3^.

Overall, our studies on C_16_-K assembly in varied ionic environments clearly demonstrate that electrostatic interactions (1) play a key role in shape selection of chiral assemblies and (2) can be systematically varied to continuously tune the nanoscale structure of the chiral assemblies. The first result is based on the observation of helicoidal scrolls in saline solutions and helices under elevated pH conditions, where the molecular degree of ionization was diminished. These observations are summarized in a structural phase diagram in Figure 4A. The second result is based on the observation that the helix radius monotonically increases with increasing pH (Table 1). We speculate that both these results can be explained by the electrostatics-driven changes in the shape of the planar membranes, from which these assemblies are derived.

### Model for Chiral Shape Selection

To explain chiral shape selection, we develop a simplified model for planar membrane energetics (eq 4, see SI, section 7) and combine it with an elementary geometric argument that helices can only be formed if the aspect ratio of the planar membranes exceeds a critical value.
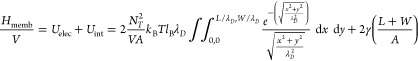
4Equation 4 describes the model for rectangular charged membranes. Here, the membrane energy density  consists of intermolecular electrostatic repulsions (*U*_elec_) and the interfacial energy (*U*_int_) due to the exposure of hydrophobic tails on the membrane edge surfaces to the aqueous solvent. In eq 4, short-ranged interactions such as intermolecular van der Waals attractions and hydrogen bonding are ignored because such interactions, while critical for assembly, do not influence the mesoscopic membrane shape. In eq 4, *N*_*T*_ is the total membrane charge. *V* and *A* are the membrane volume and surface area, respectively. *k*_B_ is the Boltzmann’s constant, *T* is the absolute temperature, and *l*_B_ and λ_*D*_ are the Bjerrum length and the electrostatic screening length, respectively. *L* and *W* are the membrane length and width, respectively. The model parameters used in the numerical calculations are listed in Table S1, SI, and the results are illustrated in Figure 4B–E.

Figure 4B shows that the membrane electrostatic energy is minimized for high aspect ratio (quasi-1D) bilayers. This is because a 1D molecular arrangement results in larger next nearest, next–next nearest neighbor distances and a smaller number of nearest, next nearest, and so on neighbors, as compared to the case of a 2D membrane. By contrast, minimization of the interfacial energy is achieved for an aspect ratio χ = *L*/*W* = 1, because for this aspect ratio, the membrane has the smallest perimeter for a fixed area (Figure 4C). Thus, the contact between the hydrophobic tails and the aqueous solvent is minimized for χ = 1. For a wide pH range, when the degree of ionization α (∝*N*_*T*_) is sufficiently high (>∼0.07), the magnitude of the electrostatic energy is greater than the interfacial energy. Therefore, the membranes are expected to exhibit a high aspect ratio (Figure 4D). At very high pH, when the vast majority of the molecules are in the deionized state, the interfacial energy dominates, and the membranes transform to sheets with χ = 1 (Figure 4D).

The above argument is consistent with the observation of helical membranes over a wide pH ∼ 6–11 range. This is because only high aspect ratio membranes, expected in this pH regime, can twist into helices. It can be readily shown that for a helix with *l* turns, the membrane aspect ratio should exceed a critical value: χ = *L*/*W* ≥ 2*l*. This relationship follows from noting that the helix pitch *P* = 2π*R* tan ψ ≥ *h* = *W*/cos ψ (Figure 3D), and the helix contour length *L* = 2π*Rl*/cos ψ. By contrast, at very high pH (≥11) the interbilayer electrostatic repulsions are very weak for the nearly deionized membranes with χ = 1, and short-ranged intermembrane attractions drive the assembly into lamellar stacks. Note that the numerical calculations in Figure 4D suggest that this transition between helical and stacked membranes is exceedingly sharp and occurs at a critical degree of ionization (or pH).

The effect of adding salt on the planar membrane shape is distinct from the above-described pH-induced changes. Experimentally, pH is increased by adding small quantities of NaOH. The number of free OH^–^ ions that can screen the membrane charge is minute and varies from 0.01 μM to 1 mM in the pH = 6–11 range. By contrast, when a few mM of salt (e.g., NaCl) is added at low pH, the range of the electrostatic interactions becomes negligible when compared to the membrane dimensions. As a result, the membrane shape becomes insensitive to the short-ranged electrostatic interactions (Figure 4E). The minimization of interfacial energy then results in planar membranes with χ = 1, even when the membranes are highly charged. These highly charged, χ = 1 membranes cannot form helices as described above. Instead, they roll into scrolls, with interbilayer separation much greater than the electrostatic screening length λ_*D*_.^15^

The above arguments suggest that the electrostatics-driven chiral shape selection between helices and cochleates should be general to crystalline charged chiral membranes. This point of view is supported by a couple of previous experimental studies. For example, crystalline membranes of a charged, chromophore amphiphile, twisted into helices and rolled into cochleates at low (1 mM) and high (50 mM) NaCl concentration, respectively.^44^ Similarly, zwitterionic phospholipid membranes forming helices^2,3,25^ and charged phospholipid membranes rolling into cochleates in solutions containing multivalent ions^12−14^ are qualitatively consistent with the idea that helices are formed when electrostatic interactions are weak, but long ranged. By contrast, cochleates are formed when the electrostatic interactions are short ranged. Thus, our theoretical model provides a simple electrostatics-based rationale for these observed transitions.

### Discussion on Helix Radius as a Function of pH

The helix radius monotonically increases with increasing pH (Table 1). At first glance, this appears counterintuitive because the strength of the membrane twisting chiral interactions relative to the electrostatic interactions is expected to increase with increasing pH. This should result in a higher curvature (smaller radius). However, note that the radius increase is concomitant with an increase in the membrane width (Table 1). Based on this positive correlation between the radius and width, and the above theoretical model, we speculate that while high aspect ratio membranes are expected in a wide pH window, both the membrane lateral dimensions increase with increasing pH. That is, the molecules can assemble into larger aggregates as the strength of intermolecular electrostatic repulsions is diminished. The increased lateral dimensions result in larger radii for helices, as more energy would be required to bend larger amounts of material. More precisely, if the chiral twisting force remains constant, we would expect a larger radius with increasing width, because the energy to form a helix from a flat membrane scales as *E*_hel_ ∝ *W*/*R*, analogous to the case for formation of a cylinder from a flat membrane.^45,46^ We also note that a positive correlation between membrane width and radius was also observed for helices in synthetic bile solutions.^17^

### pH-Dependent Assembly in C_12_-K and C_14_-K Molecular Systems

To understand how the coupling between attractive van der Waals and repulsive electrostatic interactions affects chiral assembly, we repeated pH titration and in situ SAXS measurements on aqueous dispersions of C_12_-K and C_14_-K.

Figure 2C shows the meaured titration curves for 4 mM C_*n*_-K (*n* = 12, 14, and 16) solutions along with fits based on the Hill equation (eq 1). The best fit parameters are listed in Table 2. These measurements and the analysis show that the p*K*_a_ monotonically increases with decreasing *n* (Figure 2C,D). Furthermore, the Hill parameter *m* (eq 1), while similar for C_14_-K and C_16_-K, approaches 1 (HH case) for C_12_-K (Table 2). That is reducing the number of carbons in the alkyl tails (1) enhances the propensity of the molecules in aggregates to remain ionized and (2) reduces the interdependency of molecular ionization/deionization events. Both these observations suggest that decreasing the strength of the attractive intertail van der Waals interactions, by reducing the tail length, results in aggregates with larger spacing between the charged molecular groups. This is verfied by SAXS (Figure 5A). In particular, for low pH (≪p*K*_a_), C_12_-K molecules assemble into small undefined structures or monomers because the precise shape and size of these aggregates could not be determined from the very weak SAXS signal for this sample (Figure 5A, green profile). By contrast, for C_14_-K, spherical micelles (Figure 5A, cyan profile) of radius *R*_mic_ = 2.54 nm are observed. This radius is close to the expected molecular length of C_14_-K (*t*_t_ ∼ 1.7 nm + *t*_h_ ∼ 0.75 nm). Here, 13 × 0.127 nm ∼ 1.7 nm is the expected C_14_ alkyl tail in the stretched *trans*-configuration^40^ and 0.75 nm is the expected headgroup height as derived from SAXS measurements of C_16_-K bilayers. Finally, for C_16_-K, as described earlier, ∼3.8 nm thick interdigitated, crystalline bilayers are observed (Figure 5A, navy profile). The splayed molecular arrangement in the curved spherical micelle geometry is expected to result in a larger area per headgroup than for the case of tightly packed molecules in the crystalline planar bilayer. Therefore, the SAXS-derived changes in assembly shapes with tail length are consistent with pH titration-based intuition that more “loosely” packed assemblies are formed with decreasing tail length.

**Table 2:** Best-Fit Parameters Obtained by Fitting the Titration Curves for C_*n*_-K with Hill Equation^a^

molecule	nominal [PA] (mM)	fit [PA] (mM)	p*K*_a_	*m*
C_16_-K	4.0	3.3	7.66	0.63
C_14_-K	4.0	3.2	8.01	0.60
C_12_-K	4.0	4.0	8.69	0.92

a The difference between the nominal and the fit molecular concentrations are perhaps due to errors in measuring very small quantities of flaky powder samples.

In contrast to the tail length-dependent nano/mesoscopic shapes in the pH ≪ p*K*_a_ regime, helices of crystalline bilayers are observed for all three C_*n*_-K (*n* = 12, 14, 16) molecular systems when the degree of ionization is reduced by increasing the pH. Here, the intensity profiles (Figure 5B) show the characteristic low *q* (<0.5 nm^–1^) quasi-periodic intensity modulations due to the helical structure, a broad modulation due to the bilayer thickness for 0.8 nm^–1^ < *q* < 5 nm^–1^, and diffraction peaks in the *q* > 10 nm^–1^ regime that arise from the crystalline molecular packing. Presumably, for the *n* = 12 and 14 molecular systems, the assembly into helices is preceded by the transformation of ill-defined small aggregates or micelles observed at very low pH into planar bilayer ribbons. This aspect requires further investigation as the precise transformation pathway to helices remains unclear due to the coarse pH steps in the current study. The long-term stability of helical membranes will also be investigated in our future work. This is because some previous studies^26,47^ have suggested that helical membranes with *h*/*P* < 1 are metastable intermediates to closed helices [nanotubes, *h*/*P* = 1]. However, the kinetics of the transformation from open to closed helices can be very slow (a few weeks to a few months.^26,47^). In our study, all structures were analyzed within 2 days of sample preparation, and in all cases, the helical bilayer model (eqs 2 and 3) fits to the SAXS intensity profiles (Figure 5B, red traces, and Table 3) revealed open helices. Furthermore, these fits reveal that (1) the bilayer thickness increases at the rate of ∼0.2 nm per additional carbon in the alkyl tail and (2) the helix radius increases with decreasing tail length at a fixed pH.

**Table 3:** C_*n*_-K Helix Parameters Derived from Fits in Figure 5B^a^

molecule	pH	*R* (nm)	ψ (deg)	*W* (nm)	*h*/*P*	% polydispersity
C_16_-K	6	62.6	29.3	115.6	0.59	5.5
C_16_-K	7	69.6	31.9	128.1	0.56	8.7
C_14_-K	6.5	76.1	29.0	180.45	0.68	7.4
C_12_-K	6.5	94.5	32.6	227.80	0.60	11.7

a Some bilayer parameters were fixed: *t*_h_ = 0.75 nm and ρ_h_ = 430 e/nm^3^. The best fit values for the bilayer thickness and the electron density for the hydrophobic tail region were [*t*_b_ (nm), ρ_t_ (e/nm^3^)] = [3.8, 307], [3.37, 300], and [3.0, 290] for *n* = 16, 14, and 12, respectively.

Finally, in the pH ≫ p*K*_a_ regime, where the degree of ionization, α < 0.2, assembly into multilamellar stacks is observed for all three cases (Figure 5C). Specifically, for C_12_-K, SAXS shows two strong diffraction peaks in the 1 nm^–1^ < *q* < 5 nm^–1^ range (Figure 5C, green profile). Based on fitting of the SAXS data (Figure 5C, red profile), these peaks arise due to a 1D periodic organization of the membranes in the bilayer-normal direction. For C_14_-K, the assembly consists of a mixture of helices and multilamellar stacks. This is because the SAXS intensity profile (Figure 5C, cyan profile) shows the aforementioned characteristics due to helices and the diffraction peaks due to the multilamella. For C_16_-K, the situation is like C_14_-K, with the exception that the multilamella diffraction peaks are very weak (Figure 5C, navy profile, and Figure 3C). These observations imply that at a fixed pH, the propensity for helices to transform into multilamellar stacks decreases with increasing alkyl tail length. Based on the positions *q*_1_ of the principal multilamella diffraction peak, the interbilayer spacing  are 4.49, 4.13, and 3.83 nm for C_16_-K, C_14_-K, and C_12_-K, respectively. These spacings are only 20–30% larger than the bilayer thicknesses of 3.82, 3.37, and 3.0 nm for C_16_-K, C_14_-K, and C_12_-K, respectively. This observation is consistent with the expectation that, in the very high pH regime, the interbilayer electrostatic repulsions are very weak, and short-ranged attractive interactions such as van der Waals interactions can drive the assembly into closely packed lamellar stacks.

In the medium to high pH regime two trends are puzzling. (1) At a fixed pH, the helix radius and width increase with decreasing tail length (Table 3). This is surprising because our above-described theoretical model and arguments for planar membranes suggested that the membrane width and thus the helix radius should increase with decreasing degree of ionization. Because the degree of ionization follows the sequence α_C12_ > α_C14_ > α_C16_, in the pH regime where helices are observed (Figure 2D), it was expected that the radii would follow: *R*_C12_ < *R*_C14_ < *R*_C16_. (2) It is surprising that the tendency for forming multilamella increases with decreasing tail length, in the pH 9–10 regime (Figure 5C). Based on our theoretical model, bilayer stacks are expected above a critical pH, where the vast majority of molecules are deionized, and the planar membrane transforms to the lowest perimeter (aspect ratio χ = 1) configuration. In the pH 9–10 regime, the degree of ionization is expected again to follow the trend α_C12_ > α_C14_ > α_C16_ (Figure 2D). Therefore, the fraction of stacks to helices was expected to be highest for C_16_-K and lowest for C_12_-K. These discrepancies imply that our theoretical model is very simplistic. It qualitatively explains the assembly shape selection and the nanoscale structure evolution with pH for a given molecular system but fails in explaining the quantitative trends when assembly behavior across distinct molecular systems is compared. Therefore, more detailed models or simulations are required that perhaps account for the molecular packing or the electrostatic and the steric coupling between the two leaflets of the interdigitated membrane. For example, such models may predict that bending rigidity follows the sequence κ_C12_ > κ_C14_ > κ_C16_. This could account for the observed trend in helix radius with tail length because *E*_hel_ ∝ κ. Such models and simulations are beyond the scope of the current work and will form part of our future investigations.

We note here that while helices are observed for all the three C_*n*_-K studied, the formation of these chiral mesoshapes is extremely sensitive to molecular design. If the headgroup charge is increased by adding even one additional ionizable group (e.g., C_16_-K_2_^33,34^), then only spherical and cylindrical micelles are observed over an extended pH range and some planar membranes are observed only in the regime where the degree of ionization is very low.^33,34^ For polyionic amphiphiles, helical membranes have been observed (1) for molecules that are double-tailed^26^ and (2) for single-tailed peptide amphiphiles consisting of multiple unionizable amino acids, which facilitate interheadgroup hydrogen bonding networks (e.g., β-sheet).^47,48^ Based on this observation, we speculate that single-tailed molecules with one ionizable chiral headgroup, such as C_*n*_-K, represent the simplest molecular design for analyzing chiral structures.

The pH-dependent assembly in the C_*n*_-K molecular series is summarized in Figure 5D. Most notable is the commonality that the molecules assemble into helices in the intermediate pH regime and bilayer stacks in the high pH regime when a majority of the molecules are deionized. These observations further validate the hypothesis that for crystalline membranes, helix is the equilibrium chiral morphology in the regime where electrostatic interactions are weak, but long-ranged.

### Molecular Packing via MD Simulations and WAXS

Thus, far we have focused on the meso- and nano-scale aspects of the membrane shape. Here, we combine molecular dynamics (MD) simulations (Methods, SI, section 1) and wide-angle X-ray scattering (WAXS) to investigate Å-scale molecular packing in C_*n*_-K planar membranes. In particular, we focus on molecular tilt. Theoretical models for chiral assemblies require molecules to be tilted with respect to the membrane-normal.^22−24^ However, due to orientational averaging in solution X-ray scattering and due to a limited number of diffraction peaks observed from membranes, precise determination of tilt angles from WAXS data alone is challenging. As such, combining MD simulations and WAXS analysis can prove useful in accessing information regarding molecular packing in assemblies.

We perform MD simulations on C_16_-K and C_12_-K planar bilayers as a function of degree of ionization α to understand how electrostatic and van der Waals interactions affect molecular packing. Thereafter, the molecular tilt obtained from MD simulations is used as a starting point for analysis of WAXS data for C_16_-K bilayers.

**Figure 6:**
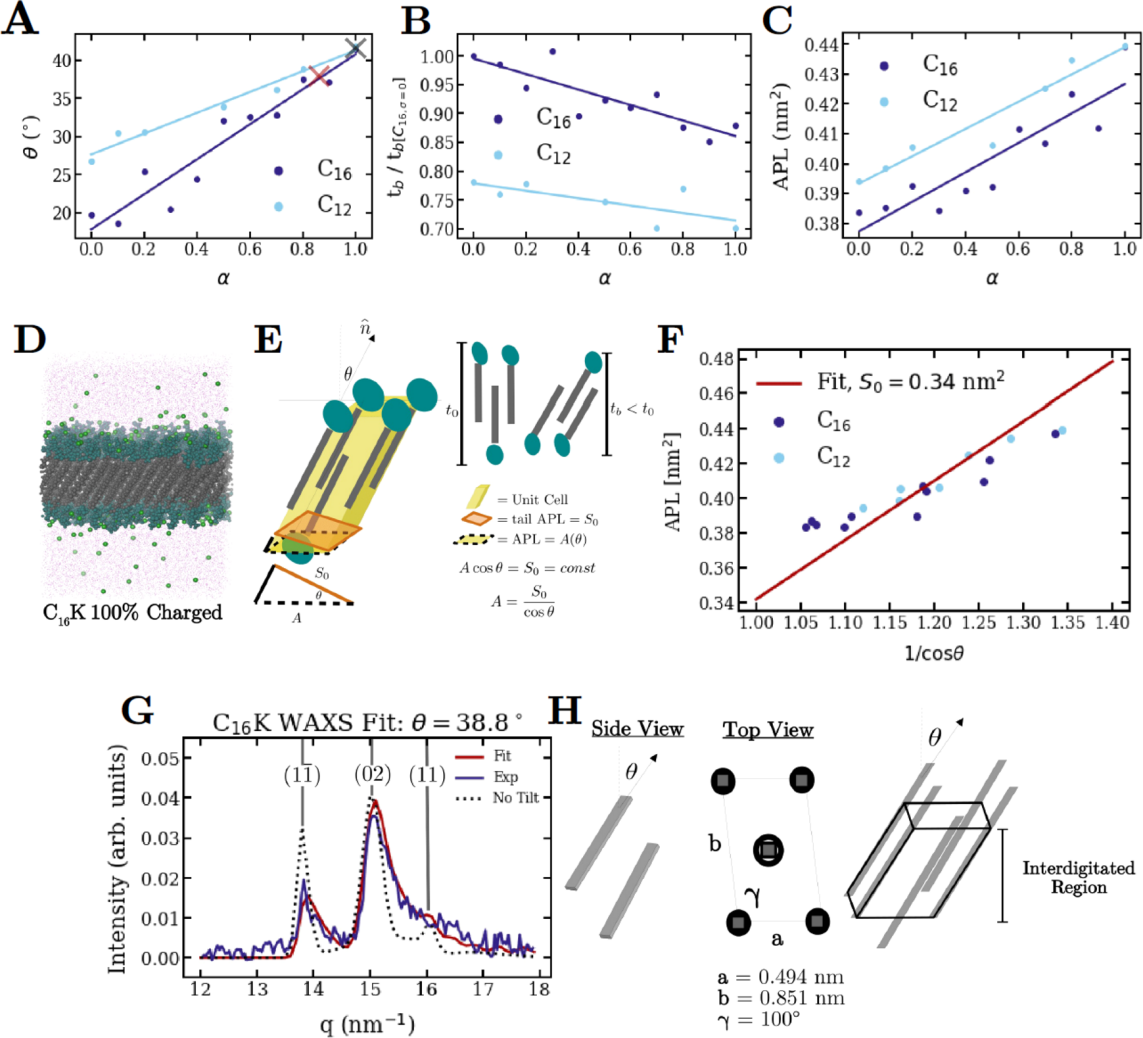
(A) Molecular tilt angle with respect to the bilayer normal, (B) bilayer thickness, and (C) area/lipid/leaflet, for C_16_-K and C_12_-K bilayers at varying degrees of ionization. Bilayer thickness in (B) is normalized to the thickness of C_16_-K bilayers at α = 0. The navy and cyan lines in (A)–(C) are guides to the eye for MD data for C_16_-K and C_12_-K bilayers, respectively. (D) Simulation snapshot of a C_16_-K bilayer after 250 ns. Purple dots: water molecules; neon green: chloride counterions; green: headgroups; gray: tails. (E) Definitions for the bilayer parameters APL, *S*_0_, θ, and *t*_b_. (F) The APL vs 1/cos(θ) data for C_16_-K and C_12_-K bilayers collapses on a straight line through origin. This demonstrates the constancy of the area per lipid tail in the tilt-normal plane. (G) WAXS data for C_16_-K bilayers at pH = 4.5 (blue) and corresponding fit (red) based on the tail packing model of the interdigitated region and oblique lattice shown in (H). Miller indices for the diffraction peaks are labeled in gray.

Figure 6 summarizes the MD simulation results. These simulations validate the X-ray scattering-derived conclusion that the molecules assemble into crystalline, interdigitated bilayers. Furthermore, consistent with theories for chiral membranes,^22−24^ the molecules are found to be tilted with respect to the bilayer-normal. These aspects are illustrated through a simulation snapshot for a C_16_-K bilayer (Figure 6D). Figure 6A–C show the MD-derived variation in important membrane parameters (defined in Figure 6E): area per lipid in a leaflet (APL), tilt magnitude (θ), and the bilayer thickness (*t*_b_) as a function of degree of ionization α, which was controlled via pH in the experiments. Figure 6C shows that APL increases with increasing degree of ionization. This is due to increased interheadgroup repulsions. Concomitantly, the alkyl tails tilt more [θ increases (Figure 6A)] such that the increase in the intertail distance is compensated. In particular, to a first approximation, the area/lipid tail/leaflet in the plane normal to the molecular tilt vector (or the molecular long axis) *S*_0_ = APL × cos (θ) is independent of α and has the same value for both C_12_-K and C_16_-K (Figure 6F). As would be expected, an increase in θ, results in a thinner membrane. That is, *t*_b_ decreases with increasing α (Figure 6B).

We note that in C_*n*_-K bilayers, APL is expected to be much larger than the cross-sectional area of an alkyl tail [∼*S*_0_/2, (Figure 6E)]. This is due to a combination of the large headgroup size and the electrostatic repulsion between these charged groups. Therefore, the packing of the alkyl tails in a single leaflet is expected to be sparse. A dense arrangement of alkyl tails can only be achieved through interdigitation. Based on this observation and the above-derived coupling between APL and θ, we speculate that maximization of tail–tail van der Waals interactions is achieved through interdigitation and molecular tilt, which minimize the intertail distances by compensating for the difference between the APL and the lipid tail cross-sectional area. These MD-derived results are qualitatively consistent with experimental studies on lipid membranes: First, the MD-derived electrostatic driven increase in APL and the constancy of the tail area in the tilt-normal plane has been previously observed in noninterdigitated lipid membranes.^49^ Second, the range of MD-derived tilt angles θ (∼18–42°, Figure 6A) is consistent with the observed tilt angles in monolayers^50,51^ and bilayers^49,52^ of double-tailed lipids for which (1) headgroup cross-sectional area is greater than that for two alkyl tails and (2) APL ∼ 0.40 nm^2^, similar to the case for C_*n*_-K (Figure 6C).

The qualitative trends in APL, θ and *t*_b_ are common to both the C_12_-K and C_16_-K bilayers. However, two tail length-dependent effects are observed. (1) For a given α, the APL (and consequently θ) is larger for C_12_-K membranes (Figures 6A,C). The equilibrium molecular distances in the membrane plane and thus the APL are determined by the competition between the attractive and repulsive intermolecular interactions. We speculate that the APL is larger for C_12_-K because the intertail van der Waals interactions are expected to be weaker than for the case of the longer tailed C_16_-K. (2) For a given α, the bilayer thickness *t*_b_ is smaller by ∼20–25% for C_12_-K (Figure 6B). This is due to the combined effects of shorter tail length and the larger molecular tilts θ for C_12_-K. However, we note that the MD results are consistent with the SAXS-derived thickness difference of ∼22% between the C_16_-K and C_12_-K membranes in the pH > 9 regime.

The above discussion shows that MD results can be rationalized by arguments based on intermolecular electrostatic and van der Waals interactions, and steric constraints on molecular packing. To test the MD results against experimental data, we analyzed the WAXS data from *C*_16_-K bilayers formed at pH ∼ 4.5 [(α ∼ 1), Figure 6G,H]. Experimentally, this is the only C_*n*_-K assembly case where unstacked or unbent bilayers were observed. Figure 6G shows the WAXS data for 13 < *q* < 17 nm^–1^, where two of the strongest diffraction peaks at *q* ∼ 13.8 and 15.0 nm^–1^ and a weak peak at *q* ∼ 16.5 nm^–1^ are observed. These peaks are due to crystalline packing of molecular tails. In our previous work,^15^ we had analyzed a similar diffraction data from a previous sample batch using a parallelepiped model for untilted tails, which were arranged on an oblique 2D lattice with *a* = 0.49 nm, *b* = 0.85 nm, and γ = 100°. Here, we show that including the tail tilt improves the fit to the data. As a starting point for WAXS analysis, we use the previously obtained lattice parameters, and the MD-simulation-derived tilt angle [θ = 41.5°, Figure 6A, black cross]. The tails were allowed to rotate about all 3 Cartesian axes. The WAXS intensity calculations powder averaged the intensities from the modeled 2D interdigitated arrangement of the parallelepiped shaped tails, following the procedure by Harutyunyan *et.al.*(53) For further details, see SI, *section 7*. The best-fit to the data is plotted in Figure 6G, and the corresponding unit cell is shown in Figure 6H. The two key findings from this analysis are as follows: (1) The oblique 2D unit cell (*u.c.*) can be described by lattice parameters *a* = 0.494 nm, *b* = 0.851 nm, and γ = 100°, very similar to those obtained via previous analysis. This corresponds to an APL = *ab* sin γ = 0.414 nm^2^/lipid/leaflet, which is very close to the MD-predicted APL for α = 1 (Figure 6C). However, we note that the above-described oblique lattice was not reproduced in our atomistic MD simulations, which showed a structure close to a hexagonal packing of molecular tails (SI, section 8). This may be due to the limited length scale (∼10.0 nm) or time scale (0.3 μs) of the simulations or limitations of the CHARMM36 force field.^54^ The limitation in achieving nonhexagonal molecular packing in MD simulations has been noted previously^55^ and requires further investigation. (2) The basis consists of two tails: one pointing downward at the *u.c.* origin and one pointing upward at the *u.c.* center. Both the tails are tilted by θ ∼ 38° with respect to the bilayer-normal. This θ matches the MD-predicted value at α = 0.9 (Figure 6A, red cross), but is slightly lower than the MD-prediction of θ ∼ 41.5° at α = 1. Nevertheless, Figure 6G shows that the tilted tail model is significantly better than the untilted tail model in describing the WAXS data. Taken together, WAXS and MD simulations show that the bilayers are interdigitated and consist of molecules that are tilted with respect to the bilayer-normal.

## Conclusions

We designed a homologous series of ionizable chiral amphiphiles C_*n*_-K (*n* = 12, 14, 16) and studied the assembly behavior of these molecules as a function of solution pH, which controlled the degree of ionization. The assembly structures were experimentally analyzed over Å to μm length scales using solution X-ray scattering and transmission electron and atomic force microscopies and were theoretically rationalized through a rudimentary model of charged membranes and MD simulations. Our multitechnique study has four key results: (1) At the Å and sub-nm scale, MD simulations in conjunction with WAXS experiments show that the C_*n*_-K bilayers exhibit crystalline packing of tilted lipid tails. The tails from the two bilayer leaflets strongly interdigitate. This packing arrangement could be explained by an interplay between van der Waals and electrostatic interactions. (2) At the mesoscale, systematic SAXS analysis showed that the crystalline, high aspect ratio membranes curve into helical bilayers in the regime where electrostatic interactions are weak, but long-ranged. In particular, helical assemblies were observed for all three C_*n*_-K molecular systems at elevated pH, where the degree of ionization was low. (3) Both MD simulations and SAXS experiments suggest that the nm-scale structure of the bilayer helices can be continuously tuned via electrostatic interactions. Particularly, the molecular tilt decreases, and helix radius increases with decreasing degree of ionization. (4) Electrostatic interactions can direct chiral shape selection: helicoidal scrolls (cochleates) are observed in saline solutions when the intermolecular electrostatic interactions are screened and short-ranged and helices are observed under conditions when the degree of ionization is low, but the electrostatic interactions are long-ranged. This finding was rationalized with an elementary theoretical model based on competition between membrane electrostatic and interfacial energies. These results highlight the versatility of our designed simple molecular systems in exploring the phase space of chiral shapes, and pave way for further studies. For example, analyzing the assemblies at higher temperatures or for molecules with shorter tails may reveal other chiral shapes such as twisted ribbons with saddle-like curvature because previous theoretical^9^ and experimental^48^ investigations suggest helix to twisted ribbon transitions in the regime where the order in the molecular packing is reduced. The experiments and simulations can be extended to analyze how membrane bending rigidities and intermolecular chiral coupling affect the nanomesoscale structure (helix and cochleate radius, helix pitch, etc.). Overall, our studies experimentally detect and explain how achiral interactions can control shape selection and nanoscale structure in chiral assemblies. These results should be useful in attaining and optimizing distinct structures based on chiral building blocks for varied applications.

## Supporting Information Available

The Supporting Information contains: . The Supporting Information is available free of charge at https://pubs.acs.org/doi/10.1021/acscentsci.2c00447.

(1) Detailed Methods. (2) Procedure for fitting titration curves. (3) Additional SAXS data for racemic mixture of C_16_-K. (4) Temperature-dependent CD study for C_16_-K. (5) SAXS simulations for helical membranes. (6) SAXS fitting procedure for helical membranes. (7) Derivation of the membrane energetics equation. (8) WAXS fitting procedure. (9) Additional discussion for MD simulation results on molecular packing (PDF)
