# Supplementary material for: Electrostatic Control of Shape Selection and Nanoscale Structure in Chiral Molecular Assemblies
Source: ACS Cent Sci. 2022 Aug 2;8(8):1169–81. doi: 10.1021/acscentsci.2c00447 (PMC9413830; doi:10.1021/acscentsci.2c00447)
Supplement: Supplementary file 1 — oc2c00447_si_001.pdf [file oc2c00447_si_001.pdf]

Supporting Information for

Electrostatic Control of Shape Selection and Nanoscale Structure in Chiral Molecular Assemblies

Joseph M. McCourt,[†] Sumit Kewalramani,[‡] Changrui Gao,[‡] Eric W. Roth, [‡] Steven J. Weigand,[§] Monica Olvera de la Cruz, [*,†,‡, ⊥] Michael J. Bedzyk[*,†,‡]

[†] *Department of Physics and Astronomy, Northwestern University, Evanston, IL, 60208, USA*

[‡] *Department of Materials Science and Engineering, Northwestern University, Evanston, IL 60208, USA*

[§] *DuPont-Northwestern-Dow Collaborative Access Team, Northwestern University Synchrotron Research Center, Advanced Photon Source, Argonne, IL 60439, USA.*

[⊥] *Department of Chemistry, Northwestern University, Evanston, IL 60208, USA*

* Correspondence: m-olvera@northwestern.edu, bedzyk@northwestern.edu

S1. Methods

The C_n-*K* assembly structures were analyzed over µm to Å-length-scales by in situ small- and wide-angle X-ray Scattering (SAXS/WAXS), atomic force microscopy (AFM), cryo-transmission electron microscopy (cryo-TEM), and circular dichroism (CD). SAXS yields structural information about the overall shape as well as membrane characteristics (radius, bilayer thickness) and WAXS provides information regarding local molecular packing. AFM and Cryo-TEM allow for visualization of the assemblies and CD provides a direct probe into the chirality of the superstructures. In addition, titration was used to determine how the protonation state of the lipid molecules changes as a function of pH. Some details are provided below.

S1.1. Peptide Synthesis. Peptides were synthesized using a CEM Liberty Blue microwave-assisted peptide synthesizer. Standard fluoren-9-ylmethoxycarbonyl (Fmoc) solid-phase peptide synthesis was used with rink amide MBHA resin (100-200 mesh). Each coupling was performed using 4 equivalents of Fmoc-protected amino acid or fatty acid, 4 equivalents of N,N'-diisopropylcarbodiimide (DIC), and 8 equivalents of ethyl(hydroxyimino)cyanoacetate (Oxyma pure) in DMF. Removal of the Fmoc groups was achieved with 20% 4-methylpiperidine in DMF and 0.1 M 1-hydroxybenzotriazole (HOBt). Peptides were cleaved from the resin using a mixture of 95% trifluoroacetic acid (TFA), 2.5% water, and 2.5% triisopropylsilane for 3 h. Crude peptide was precipitated from this solution using cold diethyl ether. Purification was carried out on a Waters Prep 150 HPLC using a water/acetonitrile with 0.1% TFA solvent system and a Phenomenex Kinetex 30x150 mm C18 column. Eluting fractions were analyzed by Electrospray Ionization Mass Spectrometry (ESI-MS) using an Agilent 6520 Q-TOF LCMS before lyophilization and purity analysis by the same Agilent LCMS (water/acetonitrile with 0.1% formic acid solvent system and Phenomenex Proteo 100 x 1 mm column).

S1.2. X-ray Scattering. SAXS/WAXS intensities ($0.02\ nm^{-1} < q < 30\ nm^{-1}$) were collected simultaneously using three Rayonix CCD detectors at the 5 ID-D undulator beamline of the Advanced Photon Source (APS) at Argonne National Laboratory. The beam size was $0.25 \times 0.25\ mm^2$, and the incident beam intensity was $\sim 3 \times 10^{11}$ photons/s. The sample solutions were injected through a capillary tube flow-cell (1.5 mm, quartz) at 2-5 mm/sec to reduce radiation damage. The

capillary was embedded in a vacuum cell to avoid air scattering. A fast shutter was used to limit sample exposure to X-rays only during the data collection time (5-10 s for each measurement). For improving statistics, 5-10 measurements were made per sample. For background subtraction and for calibrating the intensities to an absolute scale, SAXS/WAXS patterns were also collected from the empty capillary and from the capillary containing pure water before measurements on every sample. Additional SAXS/WAXS measurements were performed at 12 ID-C,D. At this APS beamline, data was collected using a Pilatus single photon counting area detector placed at a sample to detector distance (SDD) of 2.27 m. 15 or 17 keV X-ray were used with X-ray wavelength λ = 0.0827 or 0.0729 nm. The beamsize at the sample position was ~ 0.4mm × 0.2mm and the flux was 2×10^{12} photons/s. An exposure time of ~1 s was used.

The 2D SAXS/WAXS patterns were converted into 1D intensity profiles by azimuthal integration while taking into account the polarization, solid-angle and transmission corrections. The data were also normalized for the scan time. For absolute intensity calibration, the normalized and corrected scattered intensity profiles from pure water (difference of the scattering from capillary filled with water and the empty capillary) were fitted to straight lines. The intercept of these lines was set to 0.0165 cm^{-1}, the expected scattered intensity from water at $q = 0$ on an absolute scale at T = 25°C. The data from the corresponding samples were thereafter scaled accordingly.

S1.3. Cryo-Transmission Electron Microscopy. Microscopy was performed with a Hitachi HT7700 tungsten emission TEM at 100 kV and data was collected on a Gatan Orius 2 k × 4.67 k digital camera. For sample preparation, 200-mesh Cu grids with a lacey carbon membrane were glow-discharged for 30 seconds in a Pelco easiGlow glow-discharger at 15 mA with a chamber pressure of 0.24 mBar. 4uL of 4 mM amphiphile solutions were then pipetted onto the grid and plunge-frozen into liquid ethane with an FEI Vitrobot Mark III cryo plunge freezing robot with 5 seconds of blotting and a blot offset of 0.5 mm. Grids were stored in liquid nitrogen until loaded into a Gatan 626.6 cryo transfer holder cooled down to -172°C.

S1.4. Conventional Transmission Electron Microscopy. *Ex-situ* TEM imaging was also performed on a HT7700 microscope, operating at 100kV. 1% Uranyl acetate solution was used as stain to increase image contrast. A small droplet of the sample solution (10 μL) was first placed

on a TEM grid, and blotted after 3-5 min. Then a droplet of uranyl acetate solution (10 μL) was added on the grid, and blotted after 3-5 min. The grid was dried under ambient conditions for 2 h before transferring to the microscope

S1.5. Atomic Force Microscopy (AFM). Dimension FastScan AFM operating in tapping mode at 1-2 kHz frequencies was used to scan C_n-K assemblies drop-cast onto Si (1 0 0) substrates. Raw images were processed using Gwyddion. For preparing the samples, precut 1×1 cm^2 Si(1 0 0) substrates from MTI corporation were cleaned with Piranha solution (3:1 mixture of sulfuric acid and hydrogen peroxide). Substrates were then rinsed with copious amounts of ultrapure water (resistivity 18.2 MΩ・cm) and dried under a nitrogen stream. 20 μL of 4 mM amphiphile solutions were then pipetted onto the substrates and allowed to dry for 2-3 hours before AFM measurements.

S1.6. Titration. 5-6 mL solutions of 4 mM C_n-K were titrated with a 100 mM NaOH solution. The amphiphile and the NaOH solutions were freshly prepared in ultrapure water (resistivity = 18.2 MΩ・cm). The pH was measured using an Oakton pH 6 Acorn Series meter equipped with a Mettler Toledo micro electrode, which was calibrated at pH 4, 7 and 10 using buffers, prior to titration measurements.

S1.7. Circular Dichroism. 0.25-0.5 mM C_nK solutions were prepared. 400 μL of amphiphile solution was placed in a 1 mm path-length cuvette. The Jasco J-815 Circular Dichroism (CD) Spectrophotometer instrument was then used to measure the absorption spectrum of the sample. Nitrogen was flowed at a rate from 45-60 ft^3/hr (higher flow for deep UV region) to displace oxygen in the optical system such that the absorption of light by ozone formation is reduced. Raw data was processed using Spectra Manager Software.

S1.8. Molecular Dynamics Simulations. Classical all-atom explicit solvent molecular dynamics (MD) simulations were performed to study the nanoscale order of C_{12}-K and C_{16}-K flat bilayers. All simulations are performed using GROMACS 2016.3.[1] The atomistic CHARMM 36 force field (March 2019) was employed to model C_n-K bilayers, which includes Coulomb electrostatic and Leonnard-Jones interactions.[2] For simulations, all bonds containing hydrogen were constrained by the LINCS algorithm, which supported an integration time step of 2 fs. 180 lipid molecules were pre-

assembled into bilayers using the *packmol* software and solvated with TIP3P water molecules[3] and Cl^- counterions for electroneutrality. All bilayer normals were oriented in the z direction. MD simulations were carried out using a rectangular box (~ 6 × 6 × 10 nm^3) with periodic boundary conditions in x, y, z directions. The bilayer/explicit solvent system is equilibrated using the V-rescale modified Berendsen thermostat for temperature (T=300 K). Pressure equilibration used Parrinello-Rahman semi-isotropic coupling with a reference value of 1 bar, compressibility of 4.5×10^{-5} bar^{-1}, and characteristic time of 2 ps. After equilibration, *C_{16}-K* bilayer production runs were carried out in the NPT (canonical) ensemble for 250 ns with a time step of 2 fs and semi-isotropic pressure coupling with the same Parrinello-Rahman coupling parameters. *C_{12}-K* production runs were 150 ns. Periodic boundary conditions were employed in all dimensions during production simulations. Electrostatic interactions are all calculated using the particle mesh Ewald (PME) method. Real space cut-off values for electrostatic and Van der Waal forces were 1.2 nm.

S2. Fitting Titration Data with Hill Model

REACTIONS

$$\mathrm{NH_2} + (\mathrm{H^+})^m \overset{(K_a)^m}{\rightleftharpoons} \mathrm{NH_3^+}$$

$$\mathrm{H^+} + \mathrm{OH^-} \overset{K_w}{\rightleftharpoons} \mathrm{H_2O}$$

CONSTRAINTS

MASS CONSERVATION

$$C_A = [\mathrm{NH_3^+}] + [\mathrm{NH_2}]$$

CHARGE CONSERVATION

$$[\mathrm{H^+}] + [\mathrm{B^+}] + [\mathrm{NH_3^+}] = [\mathrm{OH^-}] + [\mathrm{X^-}]$$

VOLUMES

$$C_B = [\mathrm{B}] = \mathrm{V_B}\frac{\mathrm{C}_{B,init}}{\mathrm{V}_A + \mathrm{V}_B}$$

$$C_A = \mathrm{V_A}\frac{\mathrm{C}_{A,init}}{\mathrm{V}_A + \mathrm{V}_B}$$

Figure S1. *List of reactions and constraints used to define a system of equations for titration curve analysis. The Hill exponent m[4] (discussed in the main text) is included in the amphiphile headgroup protonation reaction involving $[H^+]$ and K_a. List of other variables: $K_w=10^{-14}$. C_A, amphiphile concentration; C_B, total concentration of added base (in this case, $[B^+]=[Na^+]$); V_A is the volume of the amphiphile solution before any NaOH is added and V_B is the volume of the NaOH solution added ; X^-, counterions for the protonated amphiphiles.*

The reactions, conserved quantities, and volume constraints during a titration measurement define a system of equations. We rewrite the system of equations by eliminating variables to solve for the volume of base as a function of pH (the inverse of what is experimentally performed during titration, but mathematically equivalent and more tractable). In other words, we solve this system of equations for $V_B = f([H^+]; K_a, m)$ and vary the parameters K_a, m to fit the titration data. The best-fit values for K_a and m are then inserted into the Hill equation (Eq. 3, main text) to calculate the degree of ionization at different pH levels. The above-described method for analyzing titration curves can naturally be extended to go beyond a monoprotic model [*e.g.* diprotic (H^2-A) or triprotic (H^3-A)] by including more reactions and charge/volume constraints to the system of equations.

S3. SAXS data for the C_{16}-*K* racemic mixture

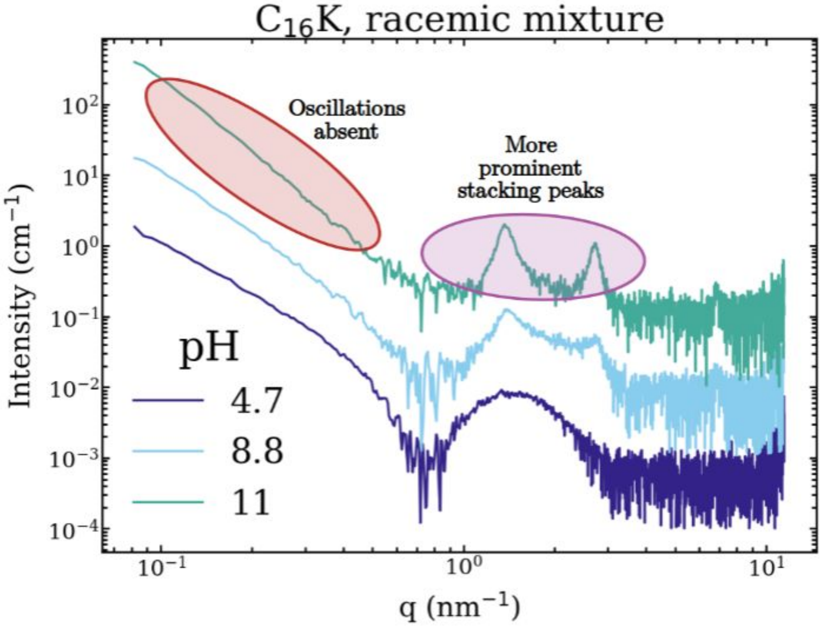

Figure S2. *SAXS intensity profiles from 4 mM dispersions of 1:1 mixture of L-C_{16}-K and D-C_{16}-K at 3 different pH. For pH = 4.7 (**Fig. S2**, bottom), the intensity profile is consistent with planar bilayers. This is similar to the L-C_{16}-K case. However, in comparison to the intensity profiles from pure L-C_{16}-K (Fig. 4A), the SAXS data from the racemic mixture at elevated pH (= 8.8, 11) do not exhibit any periodic intensity modulations in the low q (< 0.8 nm^{-1}) regime that are a signature of helical ribbons. Instead, the SAXS data from the racemic mixture shows more prominent diffraction peaks due to planar membrane stacking. These observations show that in 1:1 mixtures, the L- and D-molecules mix to form only achiral assemblies such as planar bilayers or stacks of the planar bilayers.*

S4. Temperature-Dependent Circular Dichroism

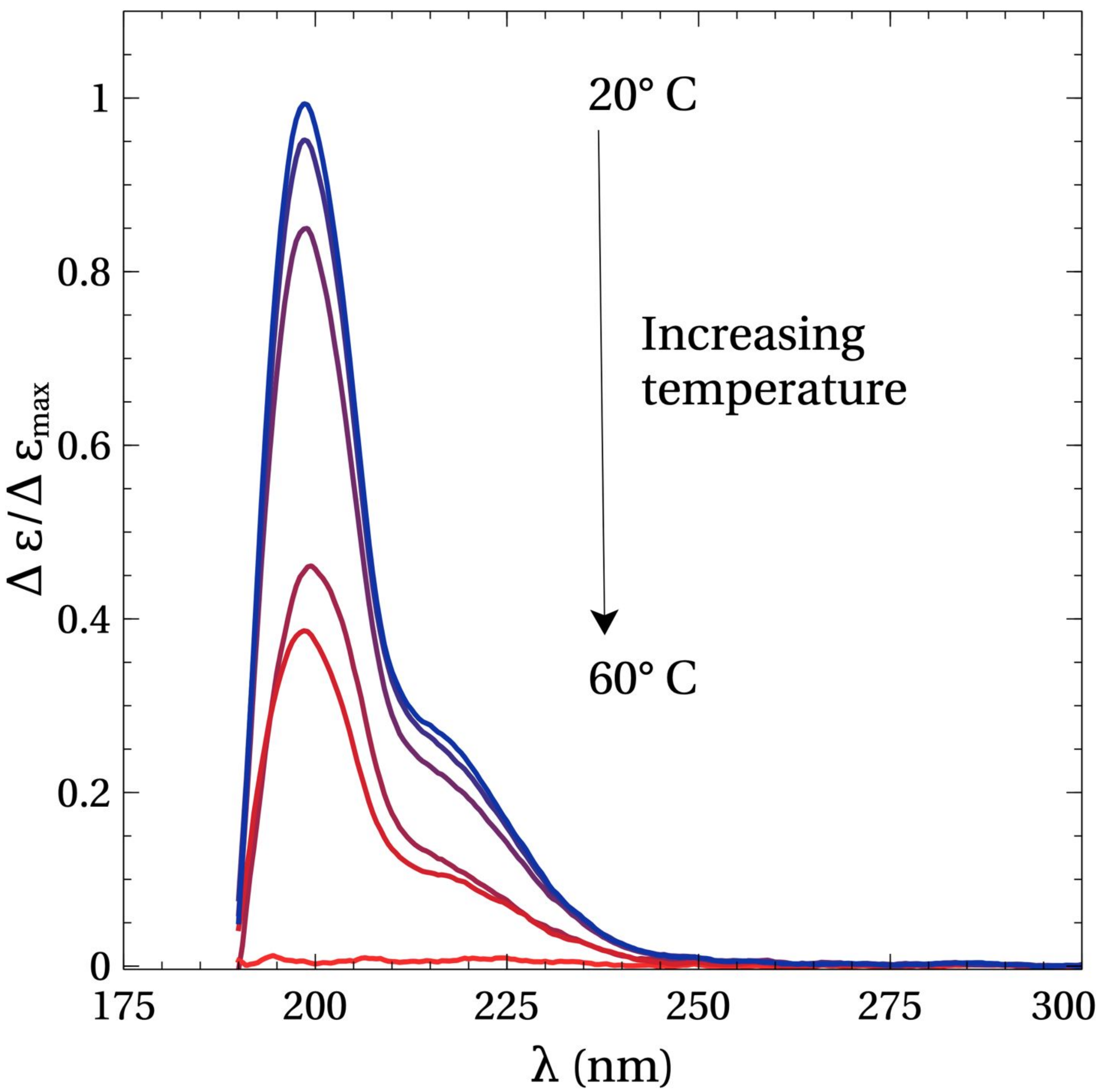

Figure S6. *Temperature-dependent CD spectra for D-C_{16}-K molecular assemblies in a solution with 10 mM NaCl. The CD signal vanishes for T = 60 °C.*

S5. Sensitivity of SAXS to Helix Parameters

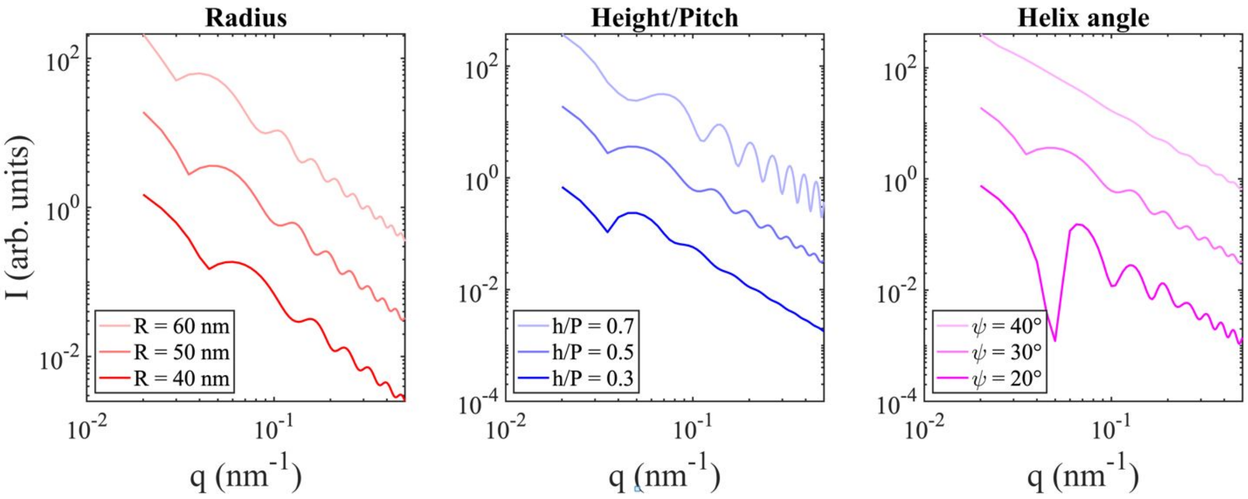

Figure S3. *Simulated intensity profiles showing the sensitivity of SAXS to parameters defining a finite width helix. Intensity profiles for 3 different Radius R at fixed height/pitch, h/P = 0.5 and a helix angle ψ = 30° (Left), 3 different h/P for fixed R = 50 nm and ψ = 30° (Center), and 3 different ψ for R = 50 nm and h/P = 0.5 (Right). For these low q simulations, the bilayer thickness was taken to be zero. We note that the finite width helix can be defined completely by 3 independent parameters such as radius R, width W and helix angle ψ. The parameter h/P was chosen instead of parameter W because it directly reflects how open is the helix. Furthermore, h/P appears as the argument of the sinc function in the helix form factor (Eq.4, main text). Based on these simulations, it is clear that the helix radius dictates the intensity modulation frequency, which increases with increasing radius (Left). This is expected based on the analogy of diffraction from circular apertures of different radii. The amplitude as well as the absolute positions of minimum and maximum in the intensity profile are modulated by h/P and ψ. The effect is coupled, but based on the above shown and other simulations it becomes clear the amplitude of the oscillations increases with h/P, that is the amount of material in the ribbon (Center). The amplitude is maximum for h/P = 1, which corresponds to the case of a nanotube.*

S6. SAXS Data Fitting Procedure

The SAXS data for helical bilayer membranes has two key features. Quasi-periodic intensity modulations in the low q (~ < 0.5 nm^{-1}) regime that arise from the helix shape and the broad intensity modulation spanning the q ~ 0.8 - 5 nm^{-1} region due to the bilayer structure of the amphiphilic membrane. The fitting of the SAXS data from helical bilayer ribbons was done in two steps.

First, the low q ($\leq$ 0.4 nm^{-1}) data was fitted using Eqs. 4 - 5 (main text) to estimate the parameters radius R, height/Pitch h/P and helix angle ψ that completely define the finite width helix. The parameter, corresponding to radius polydispersity was also optimized concurrently. This fit was done using the genetic algorithm for global optimization within the statistical toolbox of MATLAB.

Second, fitting over the extended q range encompassing the helix and bilayer intensity modulations was again performed using Eqs. 4 - 5 (main text), but for this step Trust Region Reflective algorithm for local optimization within the statistical toolbox of MATLAB was used. Here, the helix parameters were allowed to vary only by ± 15% of the best-fit values in the first step. The parameters corresponding to the bilayer were also similarly bound by constraints about the best-fit values for the electron densities and the thicknesses of the headgroup and the tail regions (ρ_h, ρ_t, t_h, t_t) obtained by fitting the SAXS data from planar membranes at low pH for C_{16}-K case. For C_{14}-K and C_{12}-K, the constraints were relaxed on the parameter t_t for the tail thickness. The two-step approach was utilized because the global optimization over the extended q-space was found to be very slow. In both the steps, the optimal parameter values were obtained by minimizing the function:

$$F = \sum_{k=1}^{n} \left[\frac{I_{meas,k} - I_{fit,k}}{I_{meas,k}}\right]^2 \quad \text{(S1).}$$

S7. Planar Membrane Energetics

The bilayer ribbon is modeled as a parallelepiped of length L, width W, and thickness δ. The membrane energy can be written as a sum of electrostatic energy and interfacial energy, which accounts for the contact between the hydrophobic chains and water on the side faces of the ribbon.

$$H_{memb} = H_{elec} + H_{int} \quad \text{(S2).}$$

The electrostatic energy is formulated as

$$H_{elec} = 2\int \rho(\boldsymbol{r})\rho(\boldsymbol{r}')u(\boldsymbol{r}-\boldsymbol{r}')d^3rd^3r' \quad \text{(S3).}$$

Here, $u(\boldsymbol{r}-\boldsymbol{r}')$ is the electrostatic potential energy for interaction between two elementary charges (here two +1 charged molecules) and is approximated as

$$u(\boldsymbol{r}-\boldsymbol{r}') \propto l_B \frac{e^{|r-r'|/\lambda_D}}{|\boldsymbol{r}-\boldsymbol{r}'|} \quad \text{(S4).}$$

Here, the Debye length □$_D \propto$ $c^{-1/2}$ and l_B is the Bjerrum length. *ρ(**r**)* the molecular charge density of the ribbon can be approximated as $N_T = \frac{\alpha M_T}{V}$, where M_T is the total number of molecules and V is the ribbon volume, and □ is the fraction of the molecules that are charged. Therefore, Eq. S3 can be rewritten as

$$H_{elec} = 2\left(\frac{N_T}{V}\right)^2 k_BTl_B\int \frac{e^{|r-r'|/\lambda_D}}{|\boldsymbol{r}-\boldsymbol{r}'|} d^3rd^3r' \quad \text{(S5).}$$

Since for C_n-*K* ribbons, only the top and bottom surfaces are charged, the volume integral can first be thought of as a 2D integral. Let $\bar{\boldsymbol{r}} = \boldsymbol{r} - \boldsymbol{r}'$ and in addition, we will perform a change of variables $x_r = \bar{x}/\lambda_D$, $y_r = \bar{y}/\lambda_D$. This results in Eq. S6,

$$H_{elec} = 2\left(\frac{N_T}{V}\right)^2 V\delta k_BTl_B\lambda_D\int_0^{W/\lambda_D}\int_0^{L/\lambda_D}\frac{e^{-\sqrt{x_r^2+y_r^2}}}{\sqrt{x_r^2+y_r^2}}dx_rdy_r \quad \text{(S6).}$$

The interfacial energy is:

$$H_{int} = 2\gamma\delta(L + W) \quad \text{(S7).}$$

In Eq. S7, γ is the surface tension at the hydrophobic tail/aqueous solution interface. The electrostatic and the interface energies in Eqs. S6-S7 when divided by the membrane volume result in energy densities in Eq. 6 in the main text.

Numerical simulations for the electrostatic and the interfacial energies as a function of salt concentration and degree of ionization (Main Text) were carried out using the following fixed parameters for the membranes:

**Paramater**	**Value**
Ribbon Area: $A = L \times W$	1 μm^2
Ribbon Thickness: δ	4 nm
Surface Molecular Density/Leaflet	2 nm^{-2}
Bjerrum Length: l_B	0.7 nm
Interfacial Tension: γ	70 mN/m

Table S1. Estimates for membrane properties. The rough estimate for the ribbon area is based on TEM images (E.g. Fig 2, Main Text), which show membranes with areas of a few μm^2*. The ribbon thickness is based on SAXS estimate of bilayer thickness of 0.38 nm. For surface molecular density, the area per lipid per leaflet is taken to be 0.5* nm^2*, which is close to the WAXS estimated APL of ~0.41* nm^2 *for* C_{16}*-K (Fig. 11, Main Text). Bjerrum length is that for pure water at T = 298 K and the hydrophobic tail/aqueous interface tension is taken to be close to that for air/water interface for simplicity.*

S8. WAXS Fitting and Analysis

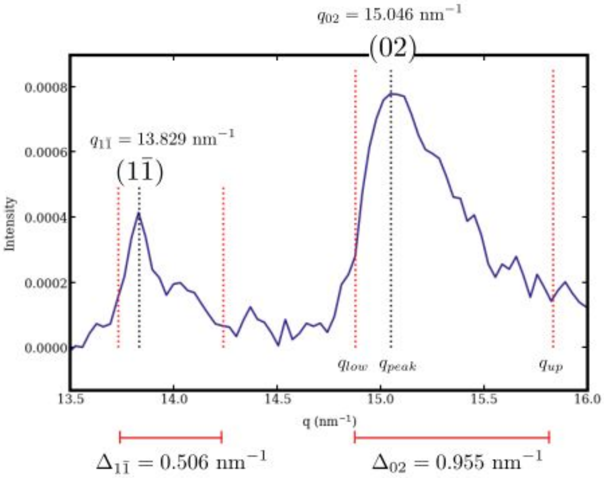

$$I(q_{low}) = \frac{1}{e} I(q_{peak})$$

$$I(q_{up}) = \frac{1}{2e} I(q_{peak})$$

$$\Delta = q_{up} - q_{low}$$

$$L_{hk} = \frac{4\lambda}{B_{hk}\cos\theta_{hk}\sqrt{\pi}} \qquad \sigma_{hk} = \frac{2\pi}{L_{hk}}$$

$$B_{hk} = \Delta_{hk} \text{ in radians}$$

$$\implies L_{10} \simeq 83 \text{ nm}, \; L_{01} \simeq 28 \text{ nm}$$

$$L_{hk} = L_{10}\left(\frac{|h|}{|h|+|k|}\right) + L_{01}\left(\frac{|k|}{|h|+|k|}\right)$$

Figure S4. *Correlation length calculation procedure based on Haruyunyan et al.[5] Peak width definitions involve an extra factor of ½ for the upper side of the peak (q_{up}) to account for the long tails of observed asymmetric diffracted intensity profiles. Peak broadening is based on the Scherrer equation and an empirical relationship for the correlation lengths of different reflections, described below.*

WAXS analysis for C_{16}-K bilayer membranes followed the procedure for 3D powder averaging of scattered intensity from a 2D lattice described by Harutyunyan *et. al.*[5] leading to an intensity profile:

$$I(q) \propto e^{-q^2\lambda_D^2} \sum_{hk} \int_0^{\pi} |F(\vec{q}_{hk}, q_z)|^2 e^{-\frac{1}{2\sigma_{hk}^2}(q\sin\theta - q_{hk})^2} \sigma_{hk} \sqrt{\frac{2\pi\sin\theta}{q q_{hk}}} d\theta \qquad \text{(S8)}$$

Here, h and k are the Miller indices for a given Bragg reflection, q_{hk} is the corresponding reciprocal lattice vector that is directly related to the real space oblique lattice parameters a, b, and γ described in the main text. $q_z = q\cos(\theta)$ is the scattering vector component normal to the 2D lattice. $F(q_{hk}, q_z)$ is the unit cell form factor resulting from interference of scattered X-rays from different molecules within a unit cell. The Gaussian term in the integrand is the effective structure factor

resulting from the summation of scattering from distinct unit cells. The leading exponential term is the effective Debye-Waller factor that arises from thermal fluctuations. The diffracted intensity is characterized by a spread □$_{hk}$ = 2□/L_{hk} that is inversely proportional to a correlation length. The correlation length for a given (h k) reflection is based on an empirical formulation: L_{hk}=L_{10}[|h|/(|h|+|k|)] + L_{01}[|h|/(|h|+|k|)]. Based on fitting of WAXS (**Fig. S4**), we find that L_{10} ~ $3L_{01}$, which we attribute to the elongated aspect ratio of the observed bilayer ribbons.

In order to calculate the unit cell form factor, we assume two C_{16}-K molecules per unit cell and focus only on the interdigitated tail region. We approximate the tails as parallelepiped rods leading to a form factor

$$F(\vec{q}_{hk}, q_z) = \rho_t \Big(\text{sinc}\big(\frac{q_x t_x}{2}\big)\text{sinc}\big(\frac{q_y t_y}{2}\big)\big(\frac{1 - e^{iq_z h_z}}{iq_z h_z}\big)\Big) \tag{S9}$$

Here the dimensions of the rod are t_x= t_y= 0.45 nm, and h_z = 1.9 nm, q_x and q_y are the x and y components of the reciprocal lattice vector q_{hk}, q_z is the z component of the scattering vector, and ρ_t is the electron density of the tails (a scaling parameter). We include the tilt of the tails with respect to the bilayer normal by performing a transformation of q_x, q_y and q_z over two rotation axes. □ is a rotation angle with respect to the x-axis and □ a rotation angle with respect to the y-axis.

$$q_{xt} = q_x \cos\beta + q_z \sin\beta \tag{S10}$$

$$q_{yt} = q_x \sin\eta \sin\beta + q_y \cos\eta - q_z \sin\eta \cos\beta \tag{S11}$$

$$q_{zt} = -q_x \cos\eta \sin\beta + q_y \sin\eta - q_z \cos\eta \cos\beta \tag{S12}$$

The tilt with respect to bilayer normal direction (z) is calculated by the dot product of the resultant rotated tail vector with the z-axis. Overall, the simulation of the scattered intensity profile was calculated by tuning the lattice and rod parameters.

S9. Molecular Dynamics Radial Distribution Functions and Lattice Structure

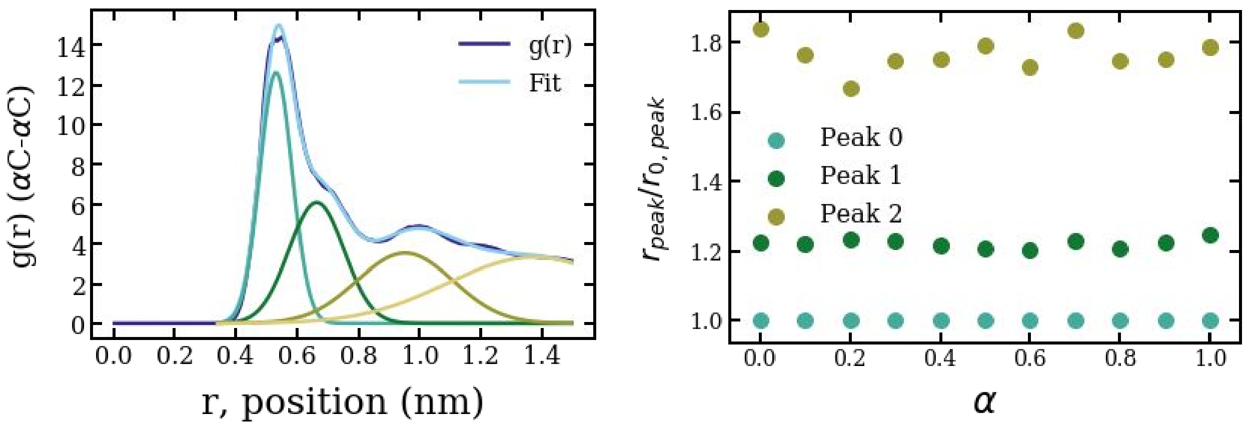

Figure S5. *Left: radial distribution function for chiral center □-Carbon – □-Carbon positions in a C_{16}-K bilayer that is derived from MD simulations of 100% charged (□ =1) membranes. Gaussian curves shown in greens and yellows are summed up for the fit to the g(r) profile. Right: relative peak positions compared to the primary peak at $r_{0,peak}$ ~ 0.53 nm for different values of □. These ratios are used to compare the observed □-Carbon lattice structure to a perfect hexagonal lattice, see below.*

Radial distribution functions were calculated for the □-Carbon locations (the chiral center of the lysine headgroup) from MD simulations for *C_{16}-K* bilayers. The distributions were fitted with gaussian peaks for systematically calculating peak positions (**Fig. S5**, left). There are 3 notable peaks within the distribution profile: a primary peak at r_0 ~ 0.53 nm, a shoulder peak at r_1 ~ 0.66 nm, and another broad peak at r_2 ~ 1 nm. The fourth gaussian peak used within the fit is not meaningful and only included to account for a background due to the semi-fluid nature of the membranes. As noted in the main texts, □-Carbon locations found in MD simulations *via* radial distribution functions do not match the lattice concluded from WAXS analysis. Instead, MD simulations suggest some degree of hexagonal order which we attribute to limitations in simulation time scales or force fields. For a perfect hexagonal lattice, the first three relative peak positions should have the following ratio relationship: $1{:}\sqrt{3}{:}2$. The positions of the broad peaks at r_0 and r_2 match the $1{:}\sqrt{3}$ ratio. However, the existence of the shoulder peak at r_1/r_0 ~1.2 is not accounted for by a pure hexagonal packing and suggests that the tilted interdigitated chains pack primarily in a distorted hexagonal lattice. These calculations were repeated for simulations at different □ values and similar distorted hexagonal packing was found for bilayers at varied degrees of ionization.

References

1. Hess, B.; Kutzner, C.; van der Spoel, D.; Lindahl, E., GROMACS 4: Algorithms for highly efficient, load-balanced, and scalable molecular simulation. *J. Chem. Theory Comput.* **2008,** *4*, 435-447.
2. Huang, J.; Rauscher, S.; Nawrocki, G.; Ran, T.; Feig, M.; de Groot, B. L.; Grubmuller, H.; MacKerell, A. D., CHARMM36m: an improved force field for folded and intrinsically disordered proteins. *Nat. Methods* **2017,** *14*, 71-73.
3. MacKerell, A. D. et al., All-atom empirical potential for molecular modeling and dynamics studies of proteins. *J. Phys. Chem. B* **1998,** *102*, 3586-3616.
4. Cantor, C. R.; Schimmel, P. R., *Biophysical Chemistry, Part III: The Behavior of Biological Macromolecules*. W.H. Freeman and Company: San Francisco, 1980.
5. Harutyunyan, B.; Dannenhoffer, A.; Kewalramani, S.; Aytun, T.; Fairfield, D. J.; Stupp, S. I.; Bedzyk, M. J., Molecular Packing of Amphiphilic Nanosheets Resolved by X-ray Scattering. *J. Phys. Chem. C* **2017,** *121*, 1047-1054.
